# Supplementary material for: Structure and catalytic regulation of Plasmodium falciparum IMP specific nucleotidase
Source: Nat Commun. 2020 Jun 26;11:3228. doi: 10.1038/s41467-020-17013-x (PMC7320144; doi:10.1038/s41467-020-17013-x)
Supplement: Supplementary file 1 — Supplementary information [file 41467_2020_17013_MOESM1_ESM.pdf]

# Supplementary Information

## **Structure and catalytic regulation of *Plasmodium falciparum* IMP specific nucleotidase**

Loïc Carrique et al.

## Supplementary Methods

### Phylogenetic analysis of ISN1 sequences

In order to generate a comprehensive database of ISN1 sequences, non-redundant entries from the Pfam protein family database (ISN1 family id: PF06437) were included. Additionally, a BLAST® search using the program BLASTp[1] was performed using *Pf*ISN1 as query sequence and significant hits not present in the Pfam database were added. Multiple sequence alignments were done using Muscle v3.8.31[2]. Alignment profiles were generated using ESPript[3]. Phylogenetic and molecular evolutionary analyses were performed with MEGA v7[4].

### *In vitro* culture of *Plasmodium falciparum*

The *in vitro* culture of the erythrocytic stages of the 3D7 strain of *P. falciparum* was maintained as described by Trager and Jensen[5]. Briefly, parasites were maintained in human erythrocytes isolated from $O^+$ blood in a culture medium containing RPMI-1640 (10.4 $g.L^{-1}$), HEPES (5.94 $g.L^{-1}$), sodium bicarbonate (0.2%), Albumax-I (0.5%) (Gibco, Life Science Technologies), hypoxanthine (0.1 mM), glucose (0.45%) and gentamycin (40 $mg.L^{-1}$). Whole blood for *P. falciparum* culturing was collected from healthy volunteers with written informed consent.

Cultures were grown in a candle jar or $CO_2$ incubator at 37 °C. For obtaining higher levels of sexual (gametocyte) stage parasites, the 3D7A cell line (MRA-154, MR4 – Malaria Research and Reference Reagent Resource Centre, Manassas, USA) was used since it has a higher sexual conversion rate. Gametocyte production and enrichment was done as described by Fivelman et al.[6]. Briefly, the method involved addition of spent medium to a synchronous culture of trophozoite stage parasites to stimulate gametocytogenesis. Further, the culture was maintained in a mixture of fresh and spent medium until the appearance of early stage

gametocytes. Enrichment of gametocytes was done by adding N-acetylglucosamine (Sigma-Aldrich) which selectively eliminates the asexual stage parasites[7].

**RNA isolation and reverse transcription-PCR of *Pf*ISN1 gene**

Total RNA was isolated from the late schizont stage of intraerythrocytic cultures of *P. falciparum* grown *in vitro* using easy-BLUE™ total RNA extraction kit (iNtRON Biotechnology, Korea) according to the manufacturer's protocol. The total RNA obtained was reverse transcribed using M-MLV (moloney murine leukemia virus) reverse transcriptase (Promega Corp., USA) and oligodT to obtain cDNA. The gene for PFL0305c (*PfISN1* gene) was amplified by PCR using this cDNA as template. Oligonucleotide primers, gene-specific FP and gene-specific RP (Supplementary Table 4) containing *Bam*HI and *Hind*III restriction enzyme cleavage sites were used. The *PfISN1* gene was cloned into a pET21bN vector containing an N-terminal hexahistidine tag. The clone pET21bN_ISN1 was confirmed by DNA sequencing.

**Expression of GFP-tagged *Pf*ISN1 in *P. falciparum***

*Pf*ISN1 coding sequence was amplified by PCR using Phusion® DNA polymerase (Thermo Fisher Scientific Inc. USA), oligonucleotide primers PfCEN FP and GFP RP (Supplementary Table 4), and pET21bN_ISN1 plasmid as template. The PCR product was cloned into pFCENv1 plasmid (kind gift from Dr. Shiroh Iwanaga, Japan,[8]) between NheI and NcoI restriction sites and the clone pFCENv1_PfISN1 was confirmed by DNA sequencing. In this construct, *PfISN1* is downstream of PbEf1α promoter and fused to GFP at the C-terminus. The PbEf1α promoter[9] is a strong constitutive promoter with high level of expression in all the asexual intraerythrocytic stages with trophozoites showing higher level of expression. The expressed protein is *Pf*ISN1 fused to GFP. The plasmid carries human DHFR gene, enabling selection on WR99210. In addition, it also carries a centromere element, enabling high efficiency

of plasmid segregation[10]. Endotoxin-free plasmid that was used for transfection was isolated using the Qiagen Endofree Plasmid Maxi kit (Qiagen) and re-suspended in sterile de-ionized water such that the final concentration of DNA was 5 µg.µL$^{-1}$.

For transfection of *P. falciparum* 3D7 strain with pFCENv1_*Pf*ISN1 plasmid, established protocols were used[11]. *P. falciparum* 3D7 was synchronized by treatment with sterile 5% sorbitol solution dissolved in sterile de-ionized water and filter sterilized through a 0.2 µm syringe filter. A 40 mL culture (6% parasitaemia, 2% hematocrit) containing mixed stages of parasites was centrifuged at 800 g. The pellet was resuspended in 5% D-sorbitol and incubated for 5 minutes, following which it was centrifuged at 800 *g* for 5 minutes and the supernatant was discarded. The cell pellet obtained was washed once with 5 mL of incomplete media, resuspended in complete media to a hematocrit of 2% and incubated in a candle jar in an incubator at 37 °C. Thereafter, the schizonts obtained from the synchronized culture were purified using Magnetic Activated Cell Sorting (MACS™) columns (Miltenyi Biotec)[12]. The Amaxa™P3 primary cell 4D-Nucleofector™ X Kit L (Lonza, Switzerland) was used for the transfection. 10 µL (50 µg) of the purified plasmid DNA was mixed with 100 µL of the P3 primary cell solution to a final volume of 110 µL. The parasites eluted from the MACS columns were harvested and resuspended in 110 µL of the P3 solution containing the plasmid DNA. Transfection was done by electroporation using 4D-Nucleofector® (Lonza, Switzerland) following which the schizonts were washed with 5 mL incomplete medium and resuspended in 2 mL of complete media at a hematocrit of 10%. The parasites were incubated in a shaker incubator at 37 °C for 2 hours, following which media was added such that the final hematocrit was 3%. Drug pressure was initiated 48 hours after transfection by the addition of 2.5 nM WR99210 (MR4 – Malaria Research and Reference Reagent Resource Centre, Manassas, USA). Medium was changed

daily. Fresh erythrocyte suspension (20 µL of 50% erythrocyte suspension) was added every alternate day. 10 days post-transfection, a small amount of culture, approximately 1 mL, was discarded once a week to prevent accumulation of old erythrocytes. The emergence of drug resistant parasites was monitored by making Giemsa-stained smears from an aliquot of the culture. 40 days post- transfection, drug resistant parasites could be observed. DNA was isolated from the parasites using standard procedures and was used for confirming the presence of the *PfISN1-GFP* gene by PCR. These confirmed lines of *P. falciparum* carrying the *PfISN1-GFP* gene were examined by live-cell fluorescence microscopy using a Zeiss® LSM-510 META confocal microscope. Images shown in Fig. 1B were processed and analyzed using the ImageJ (Fiji) software[13,14].

**Expression of GFP-tagged *Pf*ISN1 in *Plasmodium berghei***

*PfISN1* coding sequence was amplified by PCR using Phusion® DNA polymerase, oligonucleotide primers PbCEN FP and GFP RP (Supplementary Table 5), and pET21bN_ISN1 as template. The PCR product was cloned into pbCEN5 plasmid (kind gift from Dr. Shiroh Iwanaga, Japan) between *Bam*HI and *Nco*I restriction sites and the clone pBCEN5_ISN1GFP was confirmed by DNA sequencing. In this construct, *PfISN1* is downstream of PbEf1α promoter and fused to GFP at the C-terminus. The plasmid carries *Toxoplasma gondii DHFR* gene, enabling selection on pyrimethamine. In addition, it also carries a centromere element, enabling high efficiency of plasmid segregation[10]. Plasmid to be used for transfection was isolated using the QIAprep Spin Miniprep Kit (Qiagen) and re-suspended in de-ionized water.

For transfection of *Plasmodium berghei* ANKA strain with pBCEN5_ISN1GFP plasmid, established protocols were used[15]. Briefly, the protocol involved the following steps. *P. berghei* ANKA parasites were revived by injection of frozen stocks into a single BALB/c mouse and

upon reaching a parasitaemia of 30-50%, were harvested and schizonts were isolated by density gradient centrifugation on 60% Histodenz™ solution (Sigma-Aldrich). 3 µg of purified plasmid was mixed with P5 reagent (Lonza, Switzerland), added to the schizonts, and electroporated using 4D-Nucleofector®. The electroporated parasites were recovered with incomplete RPMI 1640 medium and injected into two mice through retro-orbital plexus. Parasites were seen in blood smears 4 days after injection at which point the mice were given drinking water containing the drug pyrimethamine (7 mg in 100 mL, Sigma-Aldrich) which led to the disappearance of parasites in 2 days' time. After further four days, parasites reappeared. Upon parasitemia reaching 50%, the parasites were harvested, treated with 1 X erythrocyte lysis buffer (0.15 M $NH_4Cl$, 0.01 M $KHCO_3$, 0.001 M $Na_2EDTA$; pH 7.4) for 5 min at 4 ºC and centrifuged at 500 *g* for 10 minutes. The supernatant was discarded and the erythrocyte free parasites were used for DNA isolation. DNA isolated using standard procedures was used for confirming the presence of the *PfISN1-GFP* gene by PCR using PbCEN FP and GFP RP oligonucleotide primers (Supplementary Table 5). The confirmed lines of *P. berghei* carrying the *PfISN1-GFP* gene were examined by live-cell fluorescence microscopy using a Zeiss® LSM-510 META confocal microscope. Images shown in Fig. 1C were processed and analyzed using the ImageJ (Fiji) software[13,14].

**Generation and purification of polyclonal anti-*Pf*ISN1 antibody**

Purified recombinant *Pf*ISN1 (200 µg) was emulsified with an equal volume of incomplete Freund's adjuvant and injected subcutaneously into a New Zealand White rabbit, followed by two booster doses, each 15 days apart. A small volume of blood was collected from the rabbit 15 days post second booster to check for antibody titer followed by a major bleed. The serum was separated by centrifugation after coagulation of blood and stored in multiple aliquots

at -20 °C. The polyclonal antibodies from the serum were purified by affinity chromatography using Sepharose beads conjugated to purified recombinant *Pf*ISN1. *PfI*SN1-conjugated Sepharose beads were generated using purified ISN1 and CNBr-activated Sepharose. *Pf*ISN1-specific antibodies were isolated by overnight incubation of the serum with *Pf*ISN1-conjugated beads followed by elution of the bound antibody with 50 mM glycine, pH 2.8. The eluates were collected in tubes containing 50 mM Tris-HCl, pH 8.0. The positive fractions were pooled, concentrated and stored as small aliquots at -20 °C. These were used as anti-*Pf*ISN1 antibodies.

All animal (male rabbit for anti-*Pf*ISN1 antibody generation and BALB/c strain of mice for maintenance of *P. berghei* ANKA WT and *Pf*ISN1-GFP expressing parasites) experiments adhered to the standard operating procedures prescribed by the Committee for the Purpose of Control and Supervision of Experiments on Animals (CPCSEA) and approved by the Institutional animal ethics committee (IAEC) of the Jawaharlal Nehru Centre for Advanced Scientific Research that comes under the purview of CPCSEA.

**Indirect immunofluorescence**

To check the *in vivo* localization of *Pf*ISN1 in intraerythrocytic stages of *P. falciparum*, indirect immunofluorescence microscopy was employed[16]. Briefly, the parasite culture was fixed with 4% paraformaldehyde, permeabilized with 0.1% Triton X-100 and incubated with anti-*Pf*ISN1 primary antibody (1:400 dilution), followed by Alexa®-Fluor-488-conjugated goat anti-rabbit-IgG (1:1000 dilution) (for 3D7 strain) or Alexa®-Fluor-568-conjugated goat anti-rabbit-IgG (1:1000 dilution) (for 3D7A strain) (Thermo Fisher Scientific Inc.,) secondary antibodies. Hoechst® 33342 or DAPI dye was used to stain the nucleus. Thereafter, the culture was mounted onto poly-L-lysine-coated glass slides. The images were acquired using a Zeiss® LSM META 510™ laser-scanning confocal microscope and analyzed with Zeiss® LSM Image Examiner

software and ImageJ (Fiji)[13,14]. Images shown in Fig. 1A were processed and analyzed using ImageJ (Fiji) software[13,14].

**Cloning of full-length gene, deletion and site-directed mutants of *Pf*ISN1**

Active site mutants D170N and D172N were generated by the quick-change PCR method using single mutagenic oligonucleotide primers[17] and pET-21bN_ISN1 as template. (pET-21 bN corresponds to pET-21b modified to express proteins with an N-terminal 6xHis-Tag). D170N stage I and D172N stage I primers (Supplementary Table 5) were used to insert a restriction site at the site of mutation while D170N stage II and D172N Stage II primers (Supplementary Table 5) were used to knock-out the restriction site and introduce the desired mutation at the same site. This additional step was performed to facilitate positive selection of plasmids by restriction digestion. The PCR product was digested with *Dpn*I and transformed into XL-1 blue strain of *E. coli*. The mutation site was confirmed by restriction analysis and sequencing. The plasmids were transformed into *E. coli* BL21 (DE3) strain for protein expression.

The mutants D172A, D170N-D172N, K41L, H150V, Y176L, D178V, R218L, D363V, W365L, W365Y, W365F, D367V, D394V, Q395L, F396L, H398V, D402V, F403A, F403Y, F403L, R406L and W413L as well as the deletion constructs ΔN30, ΔN30-D172N, ΔN59, ΔC10, ΔN30-ΔC10 and H150V-ΔC10 were generated using the PCR-driven overlap extension method[18]. The mutagenic/deletion fragments were then assembled into the pET-21bN plasmid by either ligation-dependent cloning or Advanced Quick Assembly (AQUA) cloning[19]. All oligonucleotide primers used are listed in Supplementary Table 5. All constructs were verified by DNA sequencing.

**Expression and purification of *Pf*ISN1 wild-type and mutant proteins for enzymatic studies**

For protein expression, either *E. coli* BL21 (DE3) or Rosetta strain were transformed

with the various plasmid constructs. A seed culture grown overnight in Terrific Broth containing the appropriate antibiotic was used to inoculate 800 mL of TB medium and induced with 0.3 mM IPTG when $A_{600}$ reached a value of 0.6. The induced culture was incubated at 18 °C for 16 h and the cells harvested by centrifugation at 4000 *g* for 10 min at 5 °C. After purification, the eluted fractions were examined by SDS-PAGE for purity. Pure protein containing fractions were pooled, concentrated, flash-frozen and stored at -80 °C. Protein concentration was estimated by the method of Bradford[20] using bovine serum albumin as the standard protein.

***Enzyme activity assays***

All experiments were done in duplicate and repeated at least three times.

**Measurement of nucleotidase activity**

Due to the difference in kinetic behaviour at low and high pH, all kinetic experiments were done at pH 5 and 8. Nucleotidase activity measurements were performed in a reaction mixture containing 50 mM Tris-HCl, pH 8.0 or 50 mM MES, pH 5.0 and 30 mM $MgCl_2$. The reaction mixture containing the substrate was mixed with 0.4 µM enzyme and incubated for a fixed time at 25 °C after which the reaction was quenched with 10% TCA. The amount of inorganic phosphate formed during the reaction was estimated by Chen's method[21]. Chens reagent (prepared fresh by mixing de-ionized water, 6N sulfuric acid, 2.5% ammonium molybdate, 10% ascorbic acid in a 2:1:1:1 ratio) was added to the quenched reaction mixture and incubated for 120 min at 37 °C. The absorbance was measured at 820 nm using a Hitachi U-2010 spectrophotometer (Hitachi High Technologies America, Inc., San Jose, CA, USA) and the amount of inorganic phosphate was calculated using a molar absorption coefficient of 25,000 $M^{-1}.cm^{-1}$. All the reaction velocities are reported as $v$/[E] in $sec^{-1}$. Substrate saturation plots for $Mg^{2}$ were obtained at pH 8.0 and pH 5.0 with the IMP concentration fixed at 60 mM and 2 mM,

respectively. To estimate the $K_d$ of ATP, the assay was done at pH 8.0 with the IMP concentration fixed at 10 mM and the ATP concentration was varied. Additional $MgCl_2$ was added to account for depletion in free $Mg^{2+}$ concentration due to the formation of ATP-$Mg^{2+}$ complex. For measurement of the pH dependence of activity, a buffer containing a mixture of 50 mM Tris-HCl, 50 mM MES and 50 mM glycine, adjusted to different pH values was used to buffer the reaction mix. Further, at each pH, activity was measured at different IMP concentrations. Initial rate *vs* substrate concentration plots were obtained in the pH range 4.0-9.0 at an interval of 1 pH unit. Data below pH 4.0 could not be obtained due to loss of enzyme stability. The data was used to obtain the pH dependence of $K_{m(app)}$ and $k_{cat(app)}$ values. To assess the magnitude of inhibition of *Pf*ISN1 activity by phosphocholine and myo-inositol-4-phosphate (Sigma-Aldrich), activity was measured in 50 mM Tris-HCl, pH 8.0, 30 mM $MgCl_2$and 5 mM IMP. The concentration of the inhibitor used was 2 mM and the assay mix containing the enzyme was incubated for 3 minutes at 25 °C. The released phosphate was estimated as described above. The control reaction contained the substrate and the inhibitor but lacked the enzyme.

**Measurement of pNPP hydrolytic activity**

Continuous measurement of pNPP (p-nitrophenyl phosphate) (Sigma-Aldrich) hydrolysis was performed by monitoring the change in absorbance due to pNP (p-nitrophenol) formation at 405 nm using a Hitachi U-2010 spectrophotometer and the UV solutions 2.1 software to collect data. Reaction mixture contained 50 mM Tris-HCl, pH 8.0 and 30 mM $MgCl_2$ and 1.5 µM enzyme, and assays were carried out at 25 °C. The amount of pNP formed was calculated using molar absorption coefficient of 18,000 $M^{-1}.cm^{-1}$. Although the mutants *Pf*ISN1D172N and *Pf*ISN1D172A were inactive on IMP, hydrolytic activity on pNPP was retained at an enhanced

level. This feature was exploited to measure the binding affinity of IMP to the mutants through inhibition of activity on pNPP. Assays contained pNPP concentration fixed at 10 mM.

**Analysis of kinetic data**

Generation of all plots and data fitting by non-linear regression were done using GraphPad Prism® version 5.0 (GraphPad Software Inc., San Diego, CA). The equations used for fitting data points in substrate saturation plots were:

**Michaelis-Menten equation** $v = (V_{\mathrm{max}}\cdot[\mathrm{S}])/(K_{\mathrm{m}}+[\mathrm{S}])$ (1),

**Simplified MWC equation** $(v/V_{max}) = (([\mathrm{S}]/K_{\mathrm{s}})(1+([\mathrm{S}]/K_{\mathrm{s}}))^{n-1})/(\mathrm{L}+(1+([\mathrm{S}]/K_{\mathrm{s}}))^{n})$ (2) [22]

and **Hill** equation[22] $v = [V_{max}\cdot \mathrm{S}^{h}]/[K_{0.5}{}^{h}+\mathrm{S}^{h}]$ (3),

where $v$ is initial velocity, $V_{\mathrm{max}}$ is maximum initial velocity, S is substrate concentration, $K_{\mathrm{m}}$ is apparent Michaelis constant, L (L = ([T]/[R])) is equilibrium constant between tensed (T) and relaxed (R) states of the enzyme, $K_{\mathrm{s}}$ is the dissociation constant of the R.S complex, $K_{0.5}$ is the apparent substrate affinity constant and $h$ is the Hill coefficient. The simplified MWC equation assumes that substrate (S) binds very weakly to the tensed (T) state of the enzyme and therefore, the dissociation constant ($K_{\mathrm{r}}$) of the T.S complex is infinite. In all cases, fits were done to multiple models and comparative statistical analysis of fit parameters was done to select the preferred model.

For determining the pH dependent ionization constant values, initial rate *vs* substrate concentration plots were obtained in the pH range 4.0-9.0 at an interval of 1 pH unit. As all plots were hyperbolic, they were fitted to Michaelis-Menten equation (eq. 1) to obtain $k_{\mathrm{cat(app)}}$ and

$K_{m(app)}$ values, which were used to obtain log ($k_{cat(app)}$) *vs* pH and log (($k_{cat}/K_m$) $_{(app)}$) *vs* pH plots. In the former plot, $k_{cat(app)}$ was independent of pH and hence the data was not fit to any equation. The data from log (($k_{cat}/K_m$)$_{(app)}$) *vs* pH plot was fitted to the following equations:

**BELL equation**: $k_{cat}/K_m$ = c/(1+H/K$_1$+K$_2$/H) (4)

**BELL equation with two ionizations on substrate**: $k_{cat}/K_m$ = c/[(1+(H/K$_1$)+(K$_2$/H)) (1+(H/K$_a$) + (K$_b$/H))] (5)

**HBBELL equation**: $k_{cat}/K_m$ = c/(1+K$_2$/H) (6)

**HBBELL equation with single ionization on substrate**: $k_{cat}/K_m$ = c/[(1+(K$_2$/H)) (1+(K$_b$/H))] (7)

where c is the pH-independent value of $k_{cat}/K_m$; H is the hydrogen ion concentration; $K_1$ and $K_2$ represent dissociation constants of the free enzyme at low and high pH, respectively; $K_a$ and $K_b$ represent dissociation constants of the substrate at low and high pH, respectively. A comparative statistical analysis of fit parameters obtained from fitting data to all the above models was done to select the preferred model.

The binding of IMP to *Pf*ISN1$_{D172A}$ and *Pf*ISN1$_{D172N}$ monitored as inhibition of pNPP hydrolysis activity was fitted to the one site binding equation,

% inhibition of $v$ = ($B_{max}$ ×$I$)/ ($K_d$+$I$)      (8)

where % inhibition of $v$ = 100 – ($v_i/v_0$) ( $v_i$ is the enzyme activity at various IMP concentrations

and $v_0$ is the enzyme activity at 0 mM IMP), $I$ is the inhibitor (IMP) concentration, $B_{max}$ is the maximum % inhibition of v, and binding constant $K_d$ is the inhibitor concentration at which half-maximum inhibition is achieved.

**Measurement of phosphotransferase activity**

To detect phosphotransferase activity in *Pf*ISN1, 5'-IMP-adenosine was used as donor-acceptor pair. The reaction consisted of 20 mM Tris-HCl, pH 8.0, 30 mM $MgCl_2$, 5 mM IMP and 5 mM adenosine, in a total volume of 100 µl.

The reaction was quenched after 5 min with 10 % TCA, neutralized with NaOH and filtered through a 0.2 µm polyvinylidene fluoride (PVDF) membrane filter. The separation of nucleotides was achieved by ion-paired reverse-phase HPLC, performed on a Genesis® C18 column (4µm pore size, 150 mm × 4.6 mm, Grace Davison Discovery Science, IL, USA), at 25 °C using an ÄKTA® Basic HPLC system (GE Amersham, UK). The mobile phase consisted of two eluants; buffer A containing 20 mM Tris-HCl, pH 7.4, 4 mM tetrabutylammonium hydrogen sulfate (TBAHS) and buffer B containing 20 mM Tris-HCl, pH 7.4, 4 mM TBAHS and 50% (v/v) acetonitrile. Prior to sample injection, the column was pre-equilibrated with several column volumes of buffer A. Post injection, the column was washed with 8.0 mL of buffer A, followed by elution of the analytes with an increasing linear gradient of buffer B in the following steps: 0-8% B in 12.0 mL; 8-15% B in 4.0 mL; 15-100% B in 8.0 mL and continued at 100% B for further 8.0 mL. Individual peaks were assigned to metabolites by running several combinations of the standard metabolites IMP, AMP, adenosine and inosine at varying concentrations under assay conditions. The flow rate was 0.45 $mL.min^{-1}$ and the analytes were detected at 254 nm. Peak integration was done using UNICORN® v3.00 software (Amersham Pharmacia Biotech AB, UK).

**Isothermal titration calorimetry**

Stoichiometry was determined for the complex of *Pf*ISN1$_{D172N}$ mutant and the ligand IMP using a VP-isothermal titration calorimeter (ITC) (Microcal, Inc., Northampton, MA, USA). The protein was extensively dialyzed against buffer containing 50 mM Tris-HCl, pH 8.0, 100 mM NaCl and 10% w/v glycerol prior to use. A stock solution of ligand IMP was made in the same buffer. Titrations were done at 25 °C by stepwise addition of small volumes (10 µL) of ligand stock (IMP at a concentration of 0.8 mM) to *Pf*ISN1$_{D172N}$ (71 µM) in the sample cell. A control experiment was performed in which the ligand at the same concentration was titrated against buffer alone in the sample cell in order to obtain the heat of dilution. The raw calorimetric signals were integrated and corrected for the heat of dilution of IMP. The resulting corrected binding isotherms were subjected to nonlinear least squares analysis using ORIGIN® software (Malvern Instruments, Malvern, U.K.) and fit to a single-site model to obtain the binding stoichiometry.

**Protein purification for structural studies**

The plasmid carrying the different constructs of *Pf*ISN1 gene was transformed into *E. coli* BL21-CodonPlus (DE3)-RIL cells. A single colony was inoculated into 10 mL of Luria Broth containing 50 µg.mL$^{-1}$ of ampicillin and grown overnight at 37 °C. The overnight culture was added to 2 L of LB with antibiotics and grown at 37 °C until the $OD_{600}$ reached ~0.6, hereafter induced with 0.3 mM IPTG (isopropyl-β-D-thiogalactopyranoside) and grown for further 18 h at 17 °C. The cells were pelleted by centrifugation at 6000 g, 10 min at 4 °C and stored at -80 °C.

The pellet was resuspended in lysis buffer (50 mM Tris-HCl pH 8.0, 100 mM NaCl - buffer A) and disrupted using a microfluidizer (3 cycles at 15,000 psi). The lysate was centrifuged at

14,000 *g*, 30 min at 8 °C and the supernatant was filtered using a 0.45 µm cutoff filter before injection onto a 5 mL HisTrap FF crude using an ÄKTA purifier (GE Healthcare). The column was washed with Buffer A containing 1 M NaCl followed by an elution in buffer A containing 500 mM imidazole. After 12% SDS–PAGE (SDS–polyacrylamide gel electrophoresis) analysis, fractions with relatively pure protein were pooled and concentrated (30 kDa cut-off Amicon Ultra-15centrifugal filter units (EMD Millipore)). Further purification by size-exclusion chromatography using a Superdex 200 10/300 GL column (GE Healthcare) was done in buffer A for preparation of samples at physiological pH and in 50 mM MES pH 5.0, 100 mM NaCl for samples at acidic pH. Aliquots of the purified protein were flash frozen and stored at -80 °C.

**Selenomethionine-derivative *Pf*ISN1 production and purification**

The plasmid carrying the *Pf*ISN1 gene was transformed into *E. coli* B834 (DE3) strain. Cells were grown overnight at 37 °C in 10 mL of selenomethionine medium base plus nutrient mix (Molecular Dimensions) supplemented with 50 µg.mL-1 L-methionine and ampicillin. The overnight culture was added to 1 L culture medium plus 50 $\mu g.mL^{-1}$ of L-methionine and ampicillin until an $A_{600}$ of 1. Cells were harvested by centrifugation at 6,000 g, 10 min at 4 °C, then resuspended in 1 L selenomethionine medium base plus nutrient mix and antibiotics without L-methionine and grown 4 h at 37 °C. Selenomethionine was added to the media at a final concentration of 50 $\mu g.mL^{-1}$ and after 30 min the culture was induced with 0.3 mM IPTG and grown for further 14 h at 17 °C. The protein was purified, concentrated and stored under similar conditions as for the unlabeled proteins.

**Size-exclusion chromatography multi-angle light scattering**

SEC-MALS experiments were performed using an analytical Superdex 200 increase 5/150 GL column (GE Healthcare Life Sciences) connected to a static light-scattering detector

(DAWN TREOS 8, Wyatt Technology) and a differential refractive index refractometer (Optilab T-rex, Wyatt Technology). 50 µL of purified sample (~13 $mg.mL^{-1}$) was injected into a column equilibrated with 50 mM Tris-HCl pH 8.0, 100 mM NaCl. Data were analyzed using the ASTRA 6.1 software package (Wyatt Technology).

**Crystallization**

Crystallization conditions screening was carried out at 292 K (vapour-diffusion in sitting-drops), using commercially available crystallization kits. For screening, a Mosquito® crystallization robot from SPT Labtech was employed using two protein/crystallization agent ratios (200 nL + 200 nL and 300 nL + 100 nL drops equilibrated against 70 µL in MRC Crystallization Plates (Molecular Dimensions)). Proteins were concentrated to 13 $mg.mL^{-1}$ in 50 mM Tris-HCl pH 8.0, 100 mM NaCl buffer except for the screen resulting in crystals at acidic pH and for which the protein was stored in 50 mM MES pH 5.0 and 100 mM NaCl. Once the crystallization conditions were established, a scale-up was performed in hanging drops mixing 2 µL protein solution with 2 µL reservoir solution (or 3 mL protein and 1 mL reservoir solution) equilibrated against 500 µL reservoir solution in 24-well plates. Crystals of selenomethionylated *Pf*ISN1 in complex with ATP were obtained in 0.1 M ammonium tartrate dibasic pH 7.0, 12% (w/v) PEG 3,350, 0.5% (w/v) n-octyl-β-D-glucoside. Crystals of *Pf*ISN1 complexed with ATP were obtained in 0.1 M sodium malonate pH 5.0, 12% (w/v) PEG 3,350, with IMP in 0.1 M HEPES pH 7.5, 0.2 M calcium acetate, 10% (w/v) PEG 8000 by co-crystallizing the protein with 5 mM of ATP and IMP, respectively. The *Pf*ISN1 apo form was crystallized in 0.1 M Tris pH 8.5, 8% (w/v) PEG 8,000. As concerns the truncated forms the crystallization conditions were 2M ammonium sulfate, 0.1M Bis-tris pH 5.5 and 0.01M urea for ΔC10; 0.1 M HEPES pH 7.5, 0.2 M calcium acetate, 10% (w/v) PEG 8000 by co-crystallizing the protein with 5 mM of IMP

for ΔN30 and 0.06M citric acid, 0.04M Bis-tris propane pH 4.1 and 16% PEG 3350 for ΔN59. Crystals were cryo-protected by adding 15% (v/v) ethylene glycol to initial conditions.

**Data collection, structure determination and refinement**

X-ray diffraction data on a selenomethionine derivative crystal (crystallized in the presence of ATP) was collected (ID29 - ESRF, Grenoble, France) at a wavelength of 0.979230 Å for SAD phasing. Phases and experimental electron density maps were calculated with the Phenix AutoSolprogramme[23] and the initial model was built using Phenix AutoBuild employing the *Pf*ISN1-ATP data for phase extension. Cycles of maximum-likelihood refinement using the program "phenix.refine"[24] were interspersed with manual corrections of the models using COOT[25]. All the remaining structures of *Pf*ISN1 were solved by molecular replacement using the ATP-bound structure as search model.

**Small angle X-ray scattering**

SAXS data were collected at the ESRF BM29 beamline in a SEC coupled mode using an analytical Superdex 200 increase 5/150 GL column (GE Healthcare Life Sciences, U.K.) equilibrated in 50 mM MES pH 5.0, 100 mM NaCl or 50 mM Tris-HCl pH 8.0, 100 mM NaCl. The injected samples were concentrated to ~13 $mg.mL^{-1}$. Initial processing was done using DataSW[26] and PRIMUS and P(r) analysis was carried out using GNOM. *Ab initio* models of *Pf*ISN1 were generated from the experimental data using both DAMMIN and GASBOR. The 50 generated models were averaged and filtered using the DAMAVER program suite to generate the final model[27] and the AllosMod-FoXS server[28].

**Negative staining electron microscopy and image analysis**

*Pf*ISN1 samples (0.025 $mg.mL^{-1}$) were applied to formvar grids and stained with 2% (w/v) sodium silicotungstate, pH 7.0. Images were recorded with a Tecnai spirit microscope

operating at 120 kV, at nominal 49,000x magnification with a pixel size of 1.36 Å. Approximately 19000 individual particles of *Pf*ISN1 were automatically picked using the LoG picker in Relion 3.0[29] before 2D classification. Good looking classes were selected and submitted to a round of 3D classification. The best-looking map containing 2,600 particles was finally refined to 20 Å resolution imposing D1 symmetry.

**Normal mode analysis (NMA)**

To predict the functional motions of *Pf*ISN1, NMA calculations were performed with the ProDy package[30].

**Comparative studies of three-dimensional structures**

Crystal structures of *Pf*ISN1 were compared with existing structures in the protein data bank at Rutgers, RCSB, using the DALI server[31].

**Figure rendering**

Figures of three-dimensional structures were drawn with PyMol (DeLano Scientific LLC, http://pymol.sourceforge.net/) and Chimera[32].

**Accession codes**

Coordinates and structure factors have been deposited in the Protein Data Bank under accession codes 6RMO (*Pf*ISN1-Apo), 6RMD (*Pf*ISN1-ATP), 6RME (*Pf*ISN1$_{D172N}$-IMP), 6RNH (*Pf*ISN1-ΔC10), 6RMW (*Pf*ISN1$_{D172N}$-ΔN30-IMP) and 6RN1 (*Pf*ISN1-ΔN59), respectively.

A

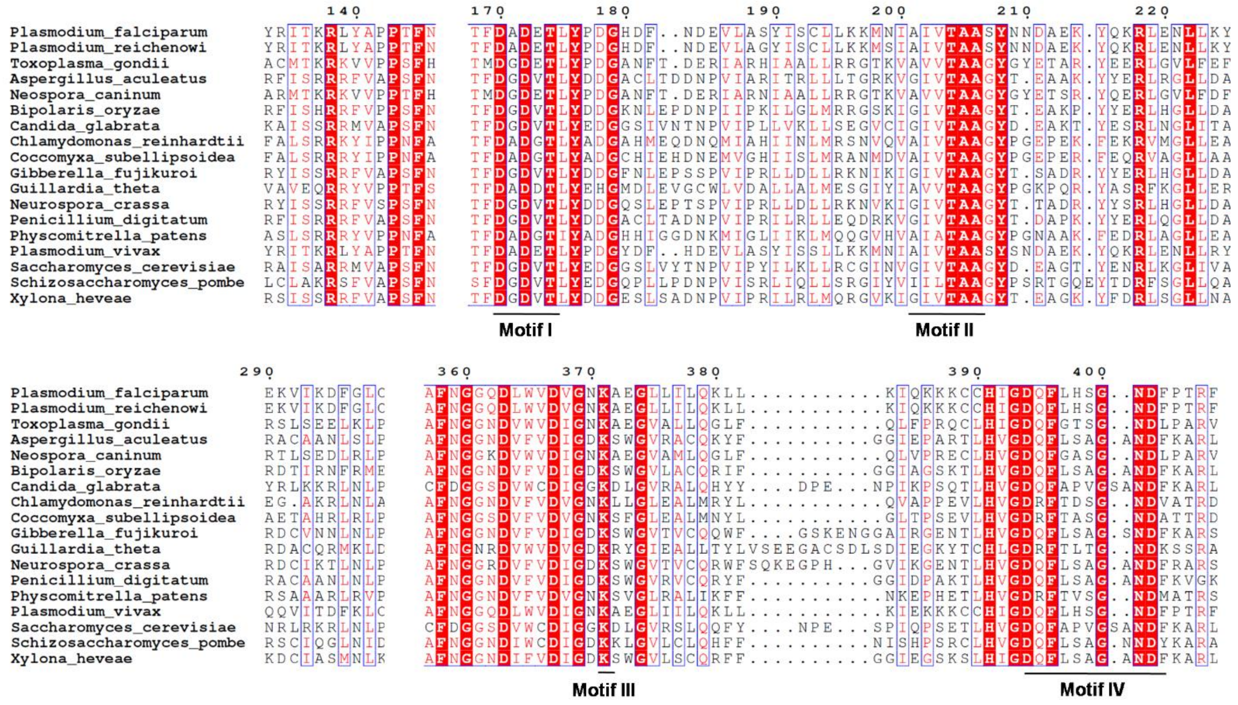


B

| Plasmodial species | Host | Gene ID |
|---|---|---|
| *falciparum* | Human | PF3D7_1206100 |
| *vivax* | Human | PVX_084340 |
| *knowlesi* | Macaque, Human | PKNA1_C2_1305800 |
| *reichenowi* | Chimpanzees | PRCDC_1205400 |
| *coatneyi* | Macaque | PCOAH_00006900 |
| *inui* | Macaque | C922_04475 |
| *malariae* | Human | PMUG01_13016100 |
| *ovale* | Human | POWCR01_130012600 |
| *gonderi** | Mandrill | GAW82794[$] |
| *cynomolgi* | Macaque | PCYB_131470 |
| *gallinaceum* | Bird | PGAL8A_00315900 |
| *relictum* | Bird | PRELSG_1304900 |
| *gaboni* | Chimpanzees | PGSY75_1206100 |
| *fragile* | Simian | AK88_02036 |
| *adleri* | Gorillas | PADL01_1206500 |
| *blacklocki* | Gorillas | PBLACG01_1205700 |
| *billcollinsi* | Chimpanzees | PBILCG01_1207100 |
| *praefalciparum* | Gorillas | PPRFG01_1248800 |

*Whole genome sequence data not available in PlasmoDB database.
[$]Accession number from NCBI protein database.

C

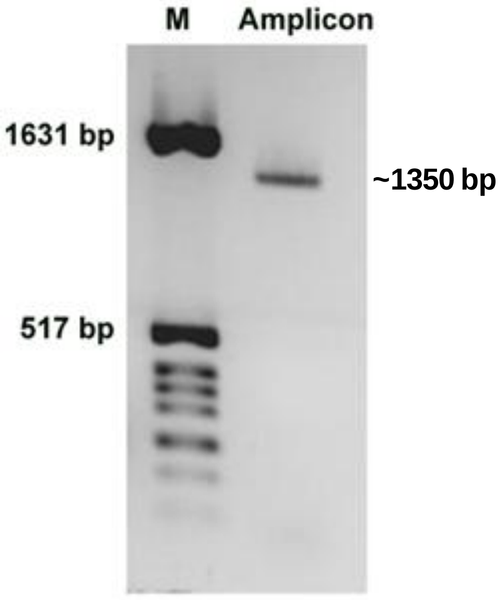

**Supplementary Figure 1: ISN1 family: sequences and expression in *P. falciparum*.**(**A**)Multiple sequence alignment of ISN1 sequences with the four HAD superfamily motifs underlined. (**B**)List of all currently sequenced Plasmodium species containing ISN1 genes. (**C**)RT-PCR on RNA isolated from trophozoite stages of intraerythrocytic *P. falciparum.* cDNA was synthesized using oligodT as primer and amplification was carried out with gene-specific FP oligonucleotide primer. The experiment was performed three times.

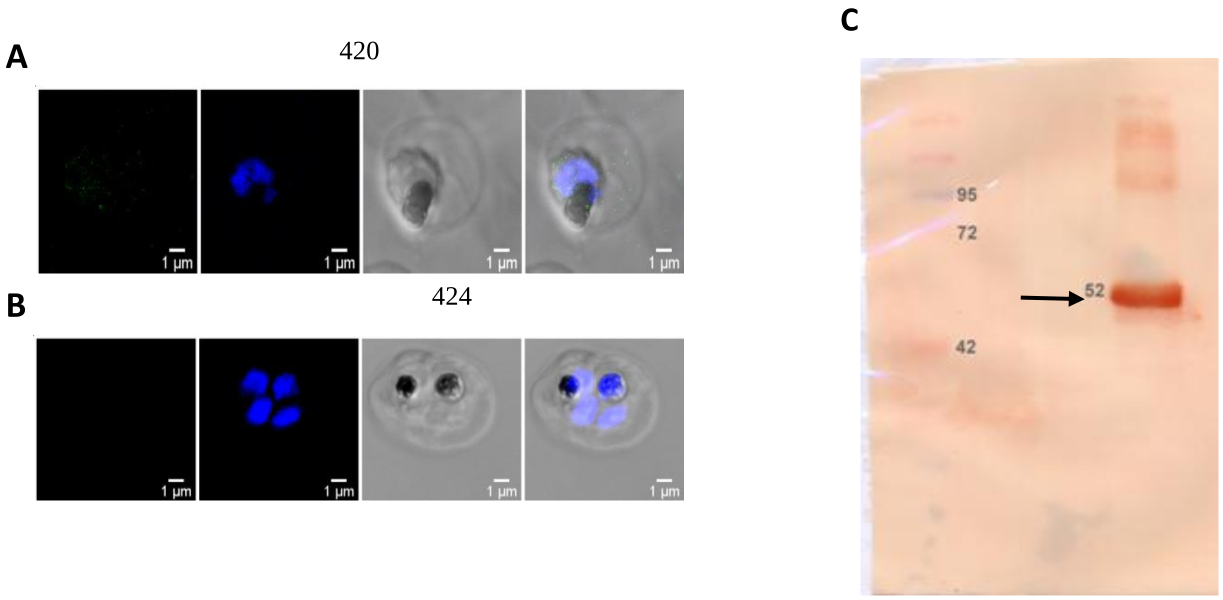


**Supplementary Figure 2: Specificity of *Pf*ISN1 antibody.** (**A**)Control with no primary (anti-*Pf*ISN1) antibody. (**B**)Control with pre-immune serum as primary antibody. Experiments in A and B were done twice (**C**)Western blot for confirmation of specificity of purified anti-*Pf*ISN1 antibody (1:1000 dilution). Lane 1: molecular mass markers (numbers indicated are in kDa), lane 2: lysate of *E. coli* cells expressing *Pf*ISN1. Blot was probed with anti-*Pf*ISN1 antibody. The band highlighted by a solid black arrow corresponds to the expected molecular mass of recombinant *Pf*ISN1. The experiment was repeated at least three times. Details of the protocol used for antibody generation and purification are provided in Methods. Source data are provided as a Source Data file.

**A**

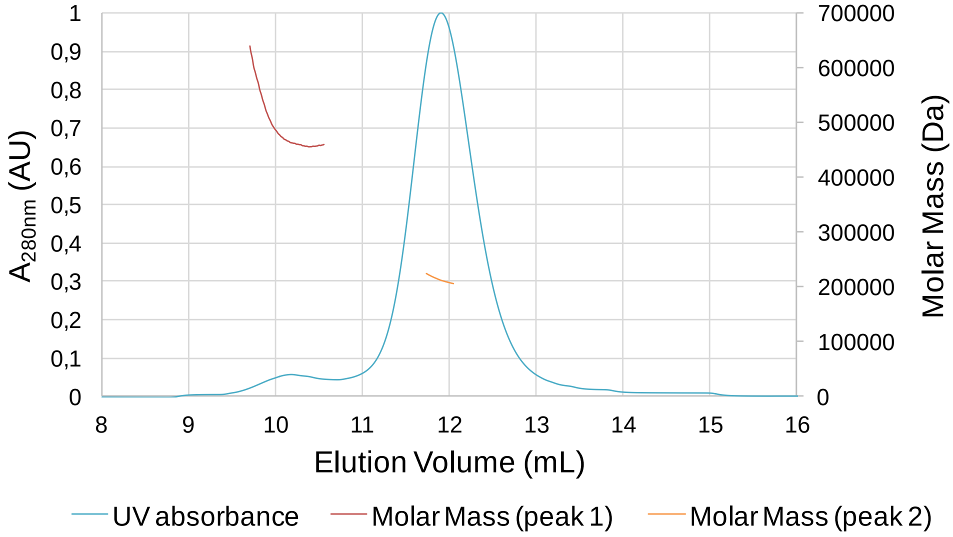


**B**

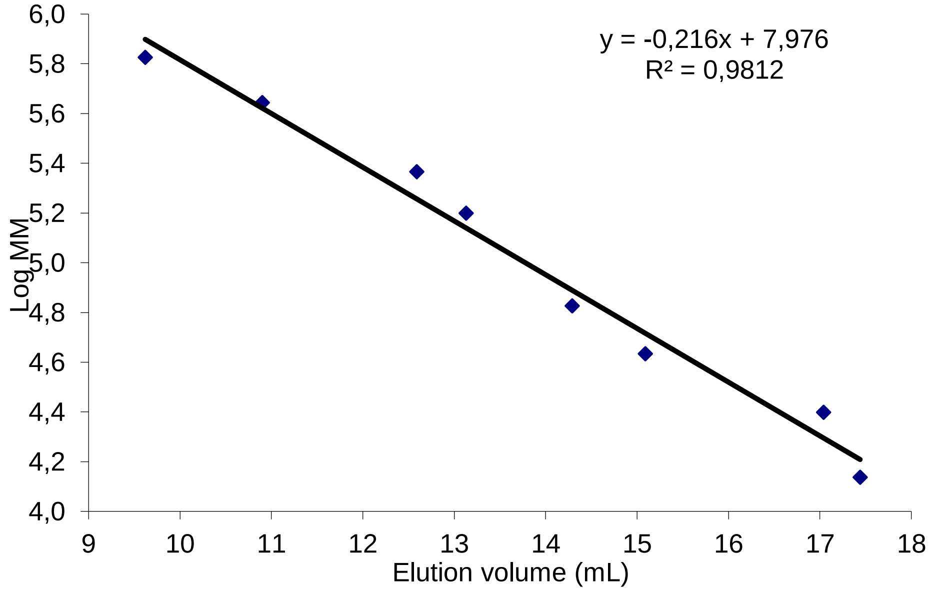


**Supplementary Figure 3: SEC-MALS analysis of *Pf*ISN1.** (**A**)Elution profiles and corresponding molecular masses determined by SEC-MALLS for *Pf*ISN1 (52 kDa for a monomer); apparent molecular mass of 593 kDa and

254 kDa for elution peaks 1 and 2 at 10.2 and 11.9 mL, respectively. (**B**)Molecular mass calibration curve obtained using thyroglobulin (669 kDa), ferritin (440 kDa), catalase (232 kDa), aldolase (158 kDa), albumin (67 kDa), ovalbumin (43 kDa), chymotrypsinogen A (25 kDa) and ribonuclease A (13.7 kDa) as molecular mass markers.

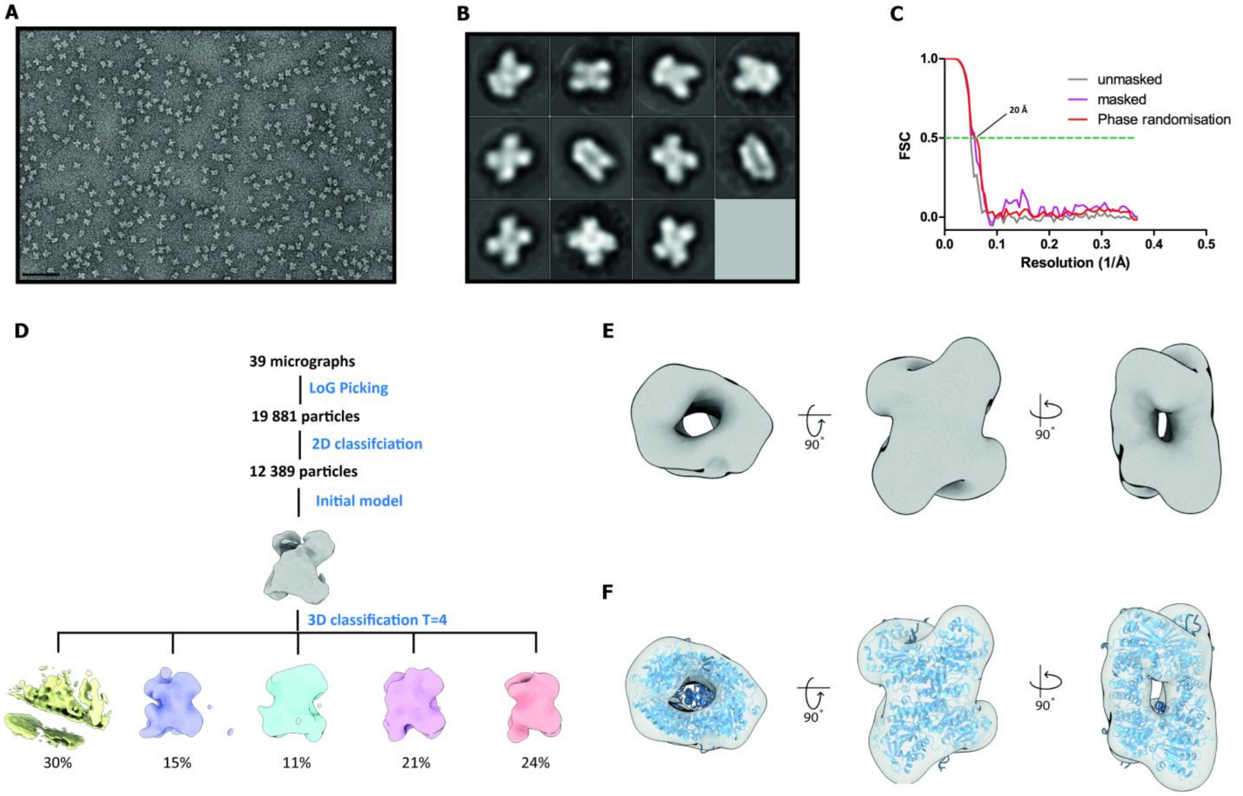


**Supplementary Figure 4: Single particles analysis of *Pf*ISN1.** (**A**)Representative micrograph of negatively stained *Pf*ISN1. Scalebar is 50 nm, and the experiment was done once. (**B**)Representative 2D class averages. (**C**)FSC curves for 3D reconstruction using gold-standard refinement in RELION, indicating overall map resolution of 20 Å at a 0.5 threshold. (**D)**Schematic representation of the processing pipeline performed in Relion[29]. (**E, F**)Overview of the final 3D reconstruction refined with D1 symmetry (**E**) and with *Pf*ISN1-Apo rigid body fitted into the density (**F**).

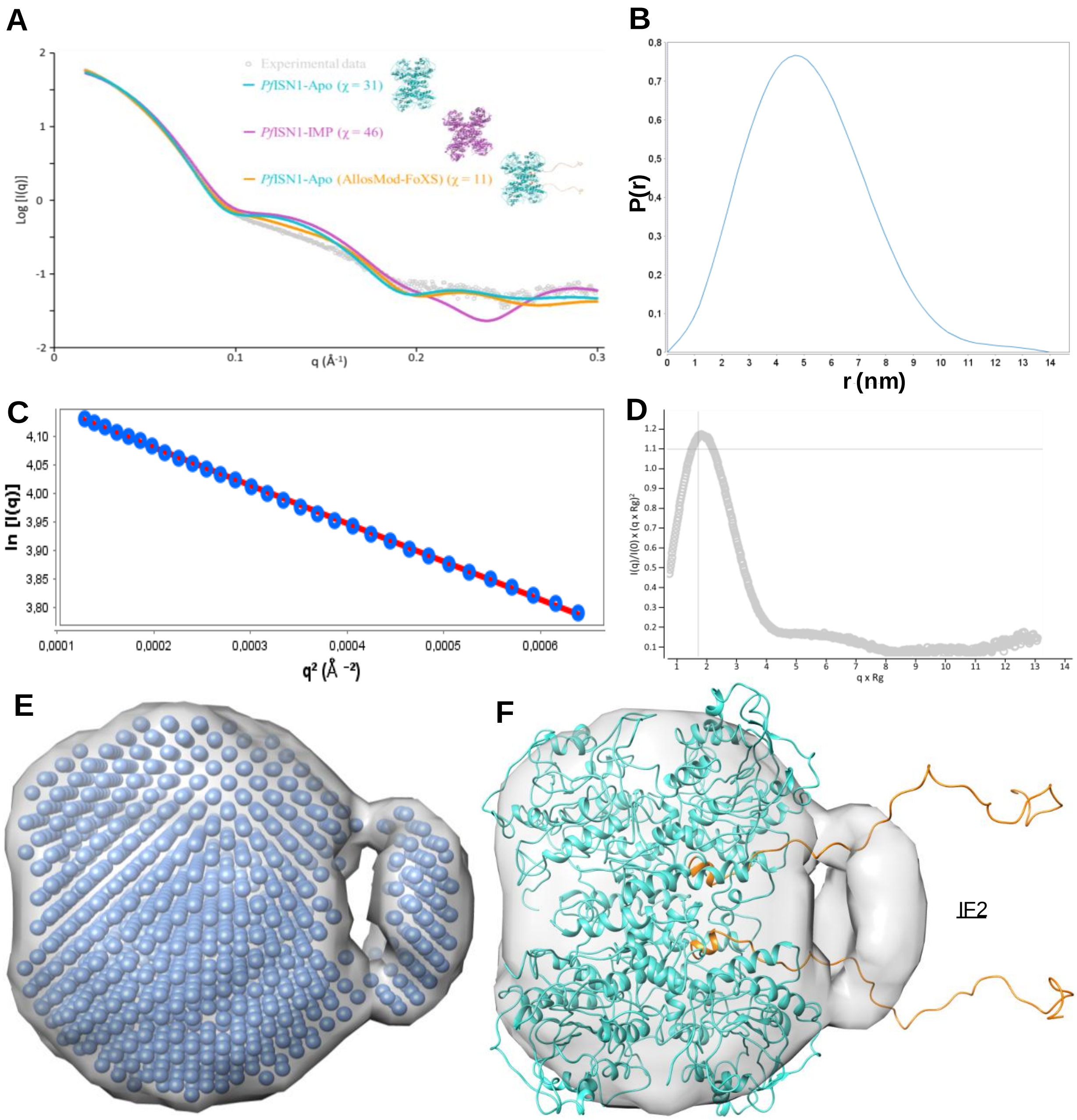


**Supplementary Figure 5: Small angle X-ray scattering studies of *Pf*ISN1-Apo.** (**A**)SAXS scattering curve of *Pf*ISN1-Apo. To compare the conformations of *Pf*ISN1 in solution and in the crystal, the scattering curve was calculated from the *Pf*ISN1-Apo structure employing the FoXS server[27], the *Pf*ISN1-IMP structure and the *Pf*ISN1-Apo structure in which the missing parts have been modelized using the AllosMod-FoXS server. The curve was fitted to the solution scattering data and the quality of the fit is expressed as X (Chi). (**B**)Distance distribution function P(r) showing a maximum dimension of approximately 140 Å. (**C**)Linear Guinier plot indicating a monodisperse protein with a radius of gyration being ~45 Å. Experimental data are in blue. (**D**)Normalized Kratky

plot calculated from the scattering data, corresponding to a well-folded protein with a tail. (**E**)*Ab initio* envelope generated using the program Gasbor[28] with blue spheres being dummy atoms. (**F**)*Ab initio* envelope in which the *Pf*ISN1-Apo structure (cyan) with missing parts modelized (orange) has been fitted. This illustrates that the missing parts are floating in the solvent at interface 2. IF2: interface 2.

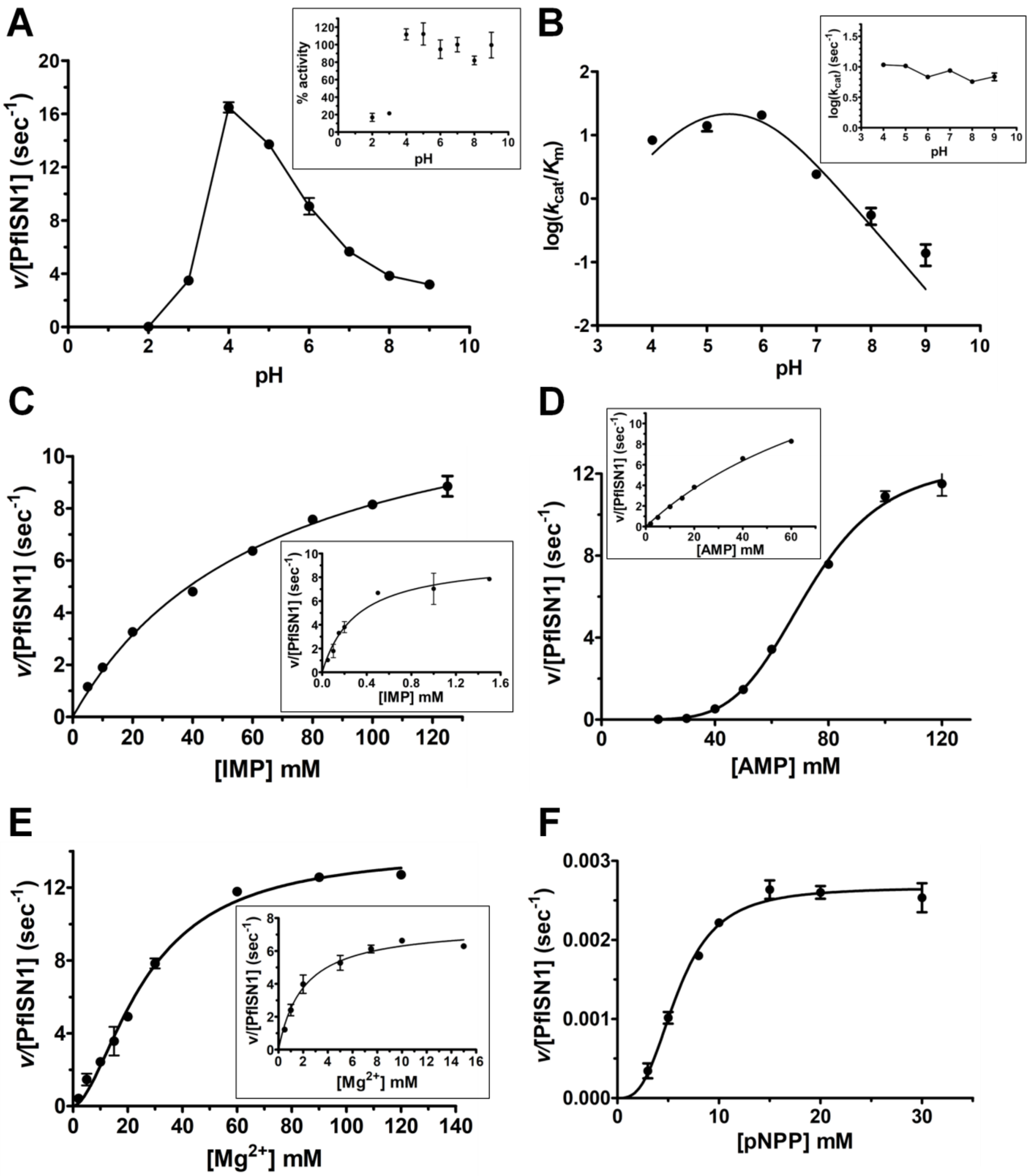


**Supplementary Figure 6: Kinetic characterization of *Pf*ISN1**. (**A**)pH-dependence of ISN1 activity. Inset shows stability of *Pf*ISN1 as a function of pH. Substrate used in both cases was IMP. (**B**)Log $(k_{cat}/K_m)_{(app)}$) *vs* pH. Line represents fit of the data to Supplementary Equation 4. Inset shows log $(k_{cat(app)})$ *vs* pH. Substrate used was IMP. (**C**)Initial rate *vs* [IMP] at pH 8.0 and pH 5.0 (inset). Line represents fit of the data to Supplementary Equation 1. (**D**)Initial rate *vs* [AMP] at pH 8.0 and pH 5.0 (inset). Lines represent fit of the data to Supplementary Equation 3 (main panel and inset). (**E**)Initial rate *vs* [$MgCl_2$], with IMP as substrate at pH 8.0 and pH 5.0 (inset). The lines represent fit of the data to Supplementary Equations 3 (main panel) and 1 (inset). (**F**)Initial rate *vs* [pNPP] plot at pH

8.0 with 30 mM $Mg^{2+}$. The line represents fit of the data to Supplementary Equation 3. Each assay was performed with two technical replicates and three independent assays were performed. In the plots data points are mean of three average values of technical replicates, each obtained from one of the three independent assays. Error bars represent standard deviation. *v*, the initial rate is the change in concentration of the product over time. Supplementary Equations 1-3 are described in Supplementary Methods. Details of equations used to select the model that best fits the data are provided in Supplementary Methods. BELL equation was the preferred model for $\log((k_{cat}/K_m))_{(app)}$ *vs* pH plot in panel B. Hill equation was the preferred model for main panels of D and E and panel F. Plots in panel C, insets in panel D and E were fitted to the Michaelis-Menten equation. Source data are provided as a Source Data file.

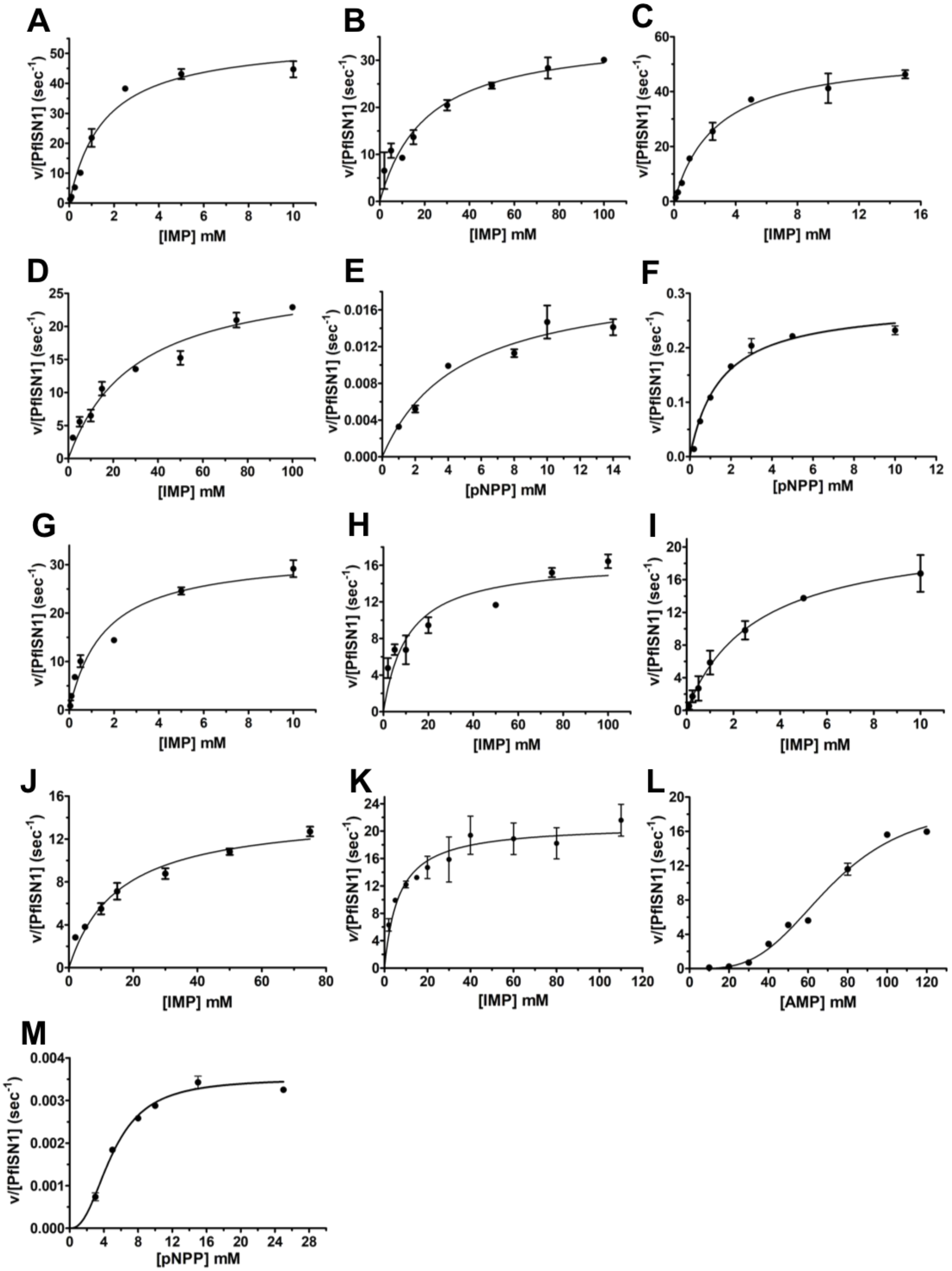

A
v/[PfISN1] (sec-1)
[IMP] mM
B
v/[PfISN1] (sec-1)
[IMP] mM
C
v/[PfISN1] (sec-1)
[IMP] mM
D
v/[PfISN1] (sec-1)
[IMP] mM
E
v/[PfISN1] (sec-1)
[pNPP] mM
F
v/[PfISN1] (sec-1)
[pNPP] mM
G
v/[PfISN1] (sec-1)
[IMP] mM
H
v/[PfISN1] (sec-1)
[IMP] mM
I
v/[PfISN1] (sec-1)
[IMP] mM
J
v/[PfISN1] (sec-1)
[IMP] mM
K
v/[PfISN1] (sec-1)
[IMP] mM
L
v/[PfISN1] (sec-1)
[AMP] mM
M
v/[PfISN1] (sec-1)
[pNPP] mM

**Supplementary Figure 7: Initial rate *vs* [S] plots for *Pf*ISN1 wild-type and mutants.** (**A**)ΔC10, pH 5.0. (**B**)ΔC10, pH 8.0. (**C**)ΔN30, pH 5.0. (**D**)ΔN30, pH 8.0. (**E**)D172A, pH 8.0. (**F**)D172N, pH 8.0. (**G**)H398V, pH 5.0. (**H**)H398V, pH 8.0. (**I**)K41L, pH 5.0. (**J**)K41L, pH 8.0. (**K, L** and **M**)wild-type activity in the presence of 4 mM ATP, pH 8.0. The substrate examined is indicated in the panels. Supplementary Equation 1 was used to fit the data in panels A-K and equation 3 was used for panels L and M. For panels A-J, each data point represents mean values +/- standard deviation of three technical replicates. For panels K-M, each assay was performed with two technical replicates. Three independent assays were performed. Each data point represents mean of three average values of technical replicates, each obtained from one of the three independent assays. Error bars represent standard deviation. *v*, the initial rate is the change in concentration of the product over time. In case of plots in panels L and M, the best fit was to the Hill equation. Source data are provided as a Source Data file.

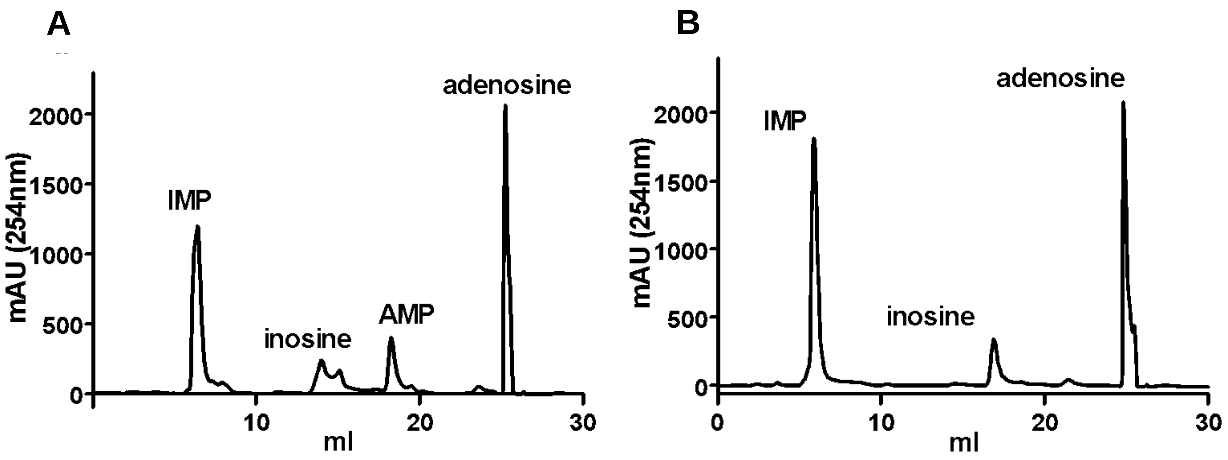


**Supplementary Figure 8: *Pf*ISN1 lacks phosphotransferase activity.** The phosphate donor-acceptor pairs used were IMP and adenosine, the activity followed by IP-RP-HPLC as described in methods. (**A**)Mixture of IMP, AMP, inosine and adenosine as standards. (**B**)Reaction mix containing 5 mM IMP, 5 mM adenosine, *Pf*ISN1 and other assay components. mAU, milliabsorbance units. Source data are provided as a Source Data file.

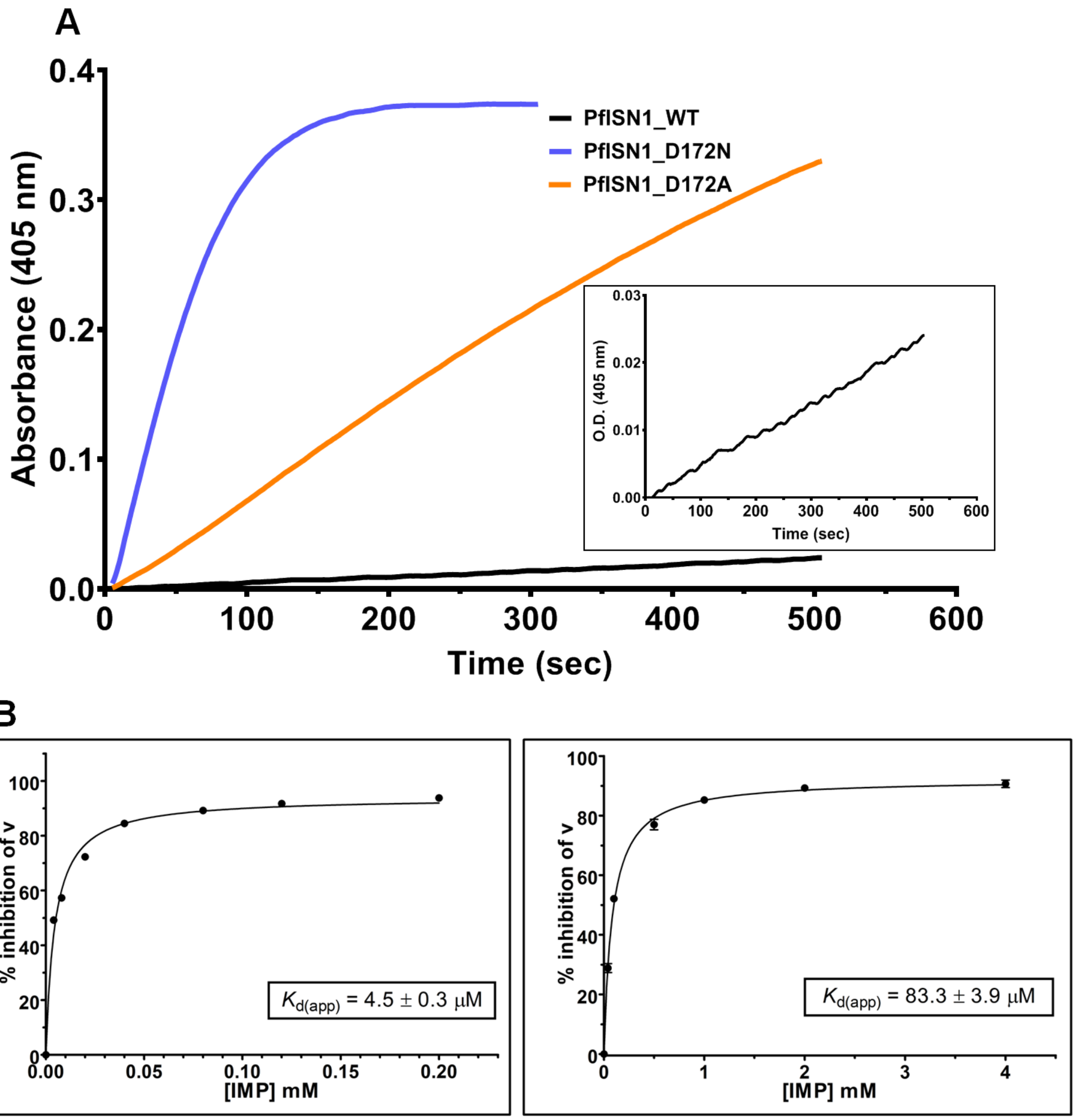


**Supplementary Figure 9: Inactive mutants, D170N and D172N of *Pf*ISN1 bind IMP tightly.** (**A**)Progress curve of pNPP hydrolysis for the wild-type enzyme and the mutants D172N and D172A. Inset: progress curve for wild-type with expanded scale for y-axis. 1.5 µM enzyme and 10 mM pNPP were used in the assays. (**B**)Inhibition *vs* IMP concentration plots for mutants *Pf*ISN1$_{D172N}$ (left panel) and *Pf*ISN1$_{D172A}$ (right panel) with their $K_{d(app)}$ values. 10 mM pNPP and 30 mM $MgCl_2$ in 50 mM Tris-HCl, pH 8.0 were used in the assay. Details of Supplementary equation 8 used to fit the data are provided in Supplementary Methods. Source data are provided as a Source Data file.

**A**

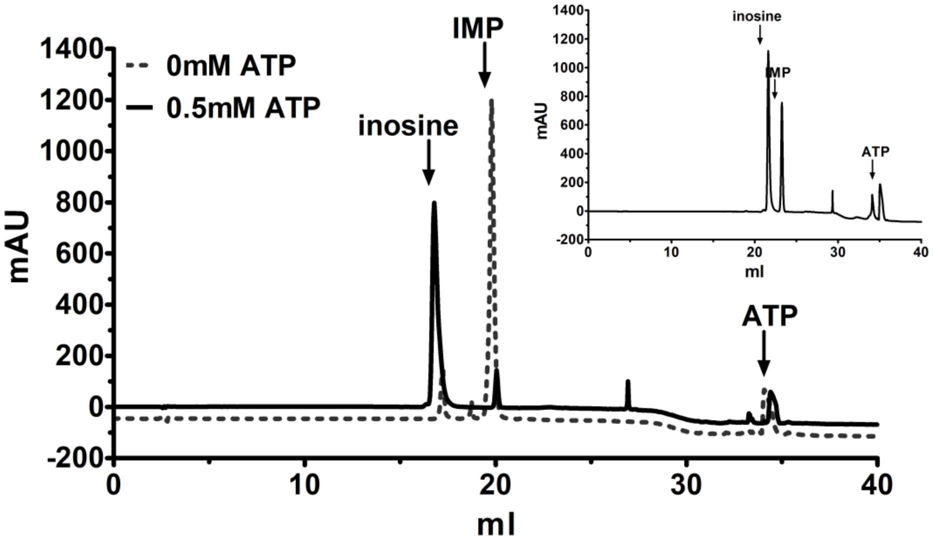


**B**

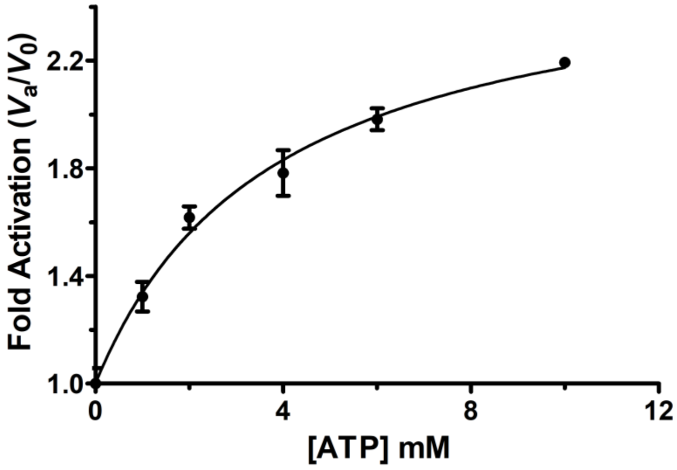


**Supplementary Figure 10: Activation of IMP-hydrolyzing activity of *Pf*ISN1 by ATP**. (**A**)Activity was followed by IP-RP-HPLC as described in Methods. The inset shows the standard elution profile of the reaction constituents (1 mM IMP, 0.5 mM inosine and 0.5 mM ATP). Absorbance was monitored at 254 nm. mAU, milliabsorbance units. (**B**)Fold activation *vs* [ATP]. IMP was fixed at 10 mM and the assay was carried out in 50 mM Tris-HCl, pH 8.0. The data were fit to one-site binding equation to obtain the $K_{d(app)}$ value. Data points are mean

with standard error of the mean as error bar. Details of the method and equation used to fit the data are provided in Supplementary Methods. Source data are provided as a Source Data file.

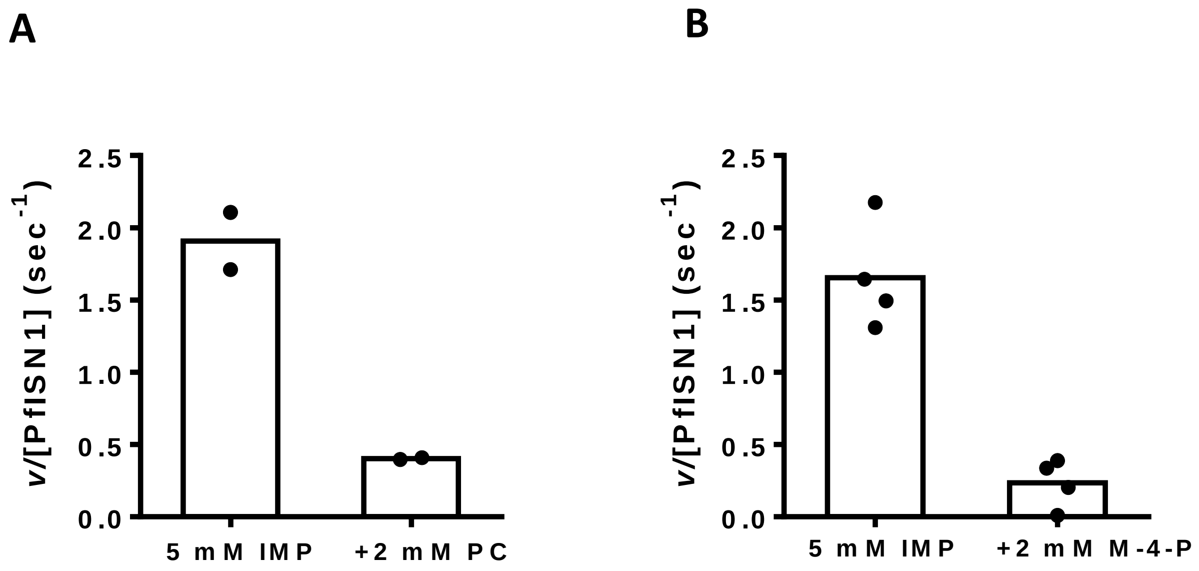


**Supplementary Figure 11: Inhibitors of *Pf*ISN1 activity. Inhibition of IMP-hydrolyzing activity of *Pf*ISN1 by (A)**phosphocholine (PC) and **(B)**D-myo-inositol-4-phosphate (M4P). Both assays were performed in duplicate and repeated twice. The values in the plots are mean with the error bar denoting standard error of the mean. *v*, the initial rate is the change in concentration of the product over time. The conditions used for the assays are as mentioned in the Methods section.

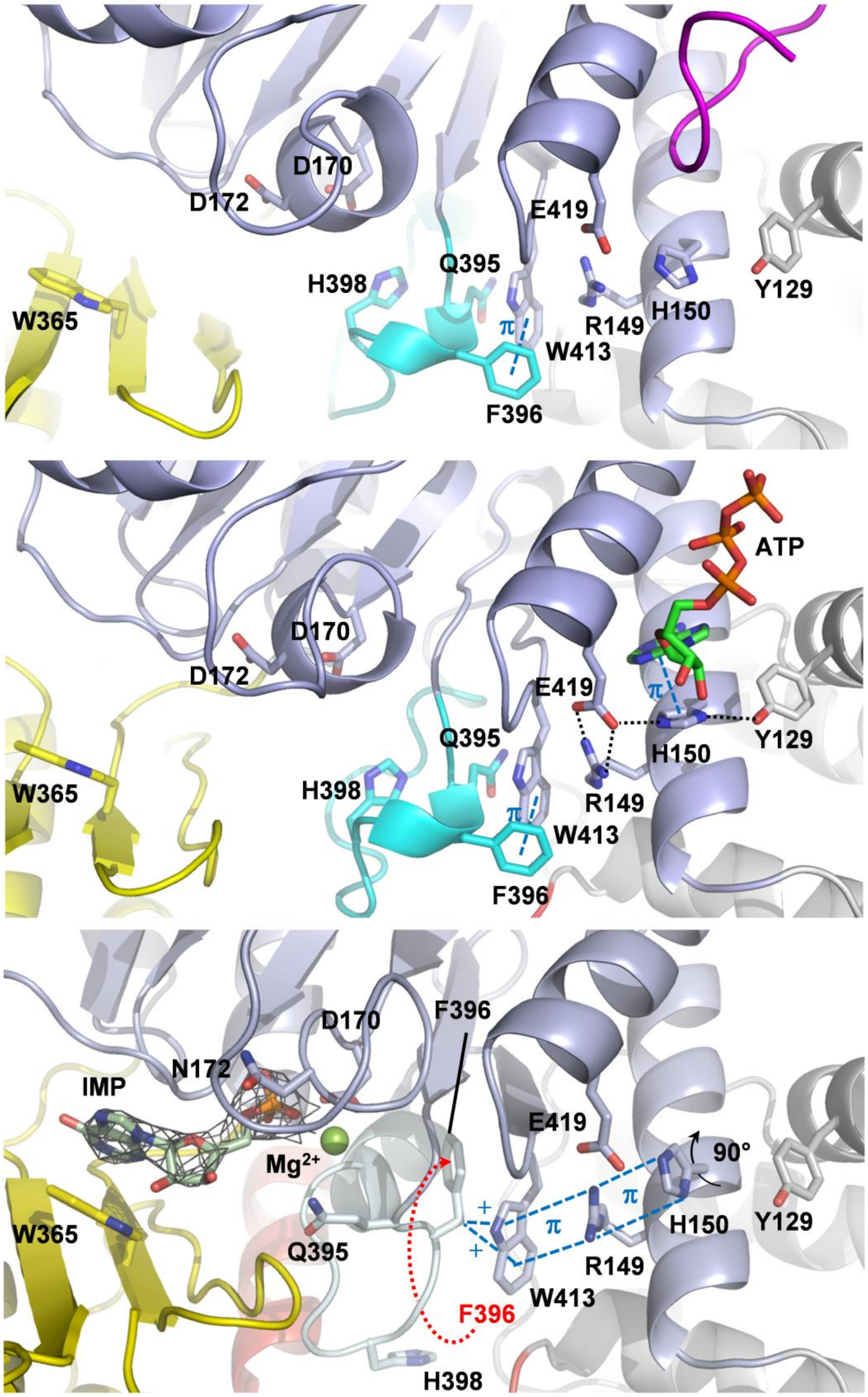


**Supplementary Figure 12: Long distance interactions.** (**A**)In *Pf*ISN1-Apo, a unique π-stacking is observed between F396 and W413 (**B**)In *Pf*ISN1-ATP, the nucleotide forms a π-stacking with H150 which is stabilized by a

hydrogen bond with Y129 and a salt-bridge with E419 (**C**)In *Pf*ISN1$_{D172N}$-IMP, the reorganization of Segment$_{D394-F407}$ induces a long distance interaction with the effector site, but also H150 rotation and ATP release.

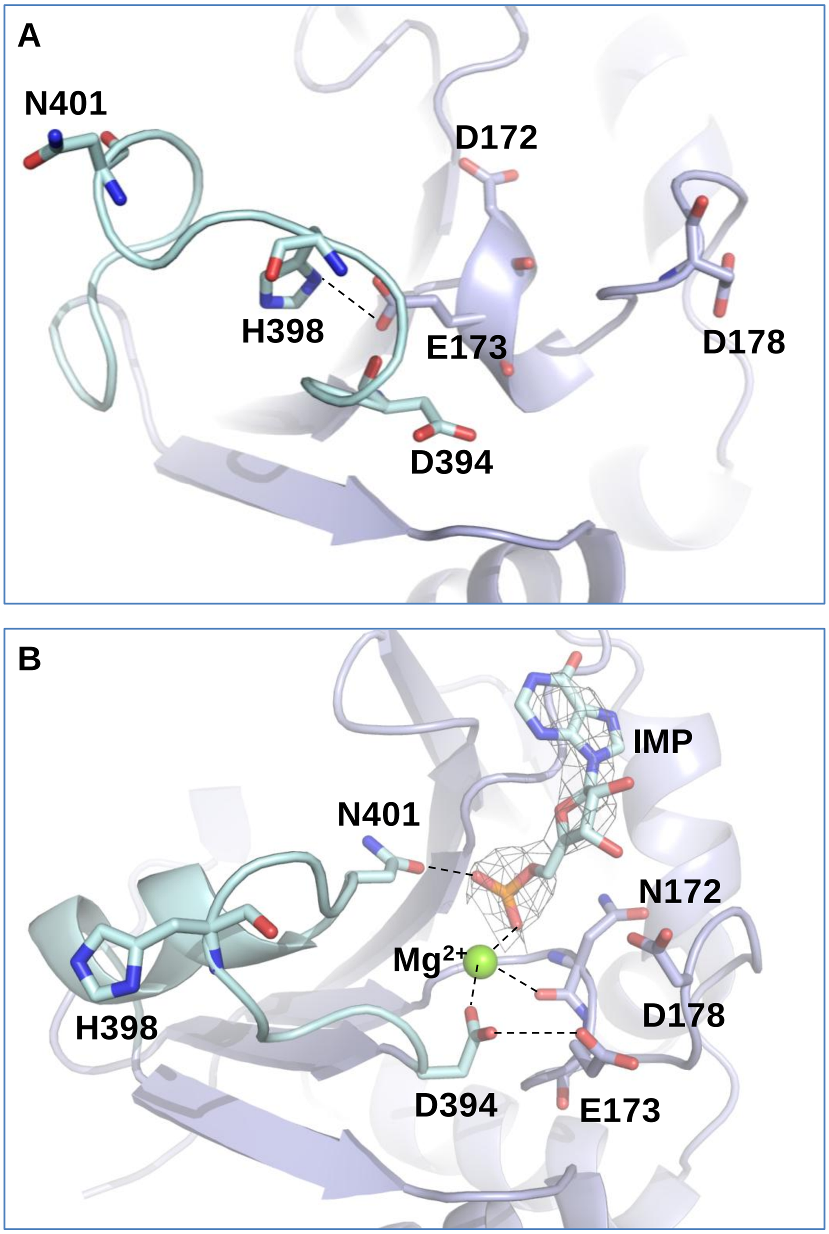


**Supplementary Figure 13**: **Reorganization of Segment$_{\mathbf{D394\text{-}F407}}$** (depicted in cyan) (**A**)in apo- and (**B**)in IMP-bound conformations. *2Fo-Fc* density corresponding to IMP has been contoured at 1 σ.

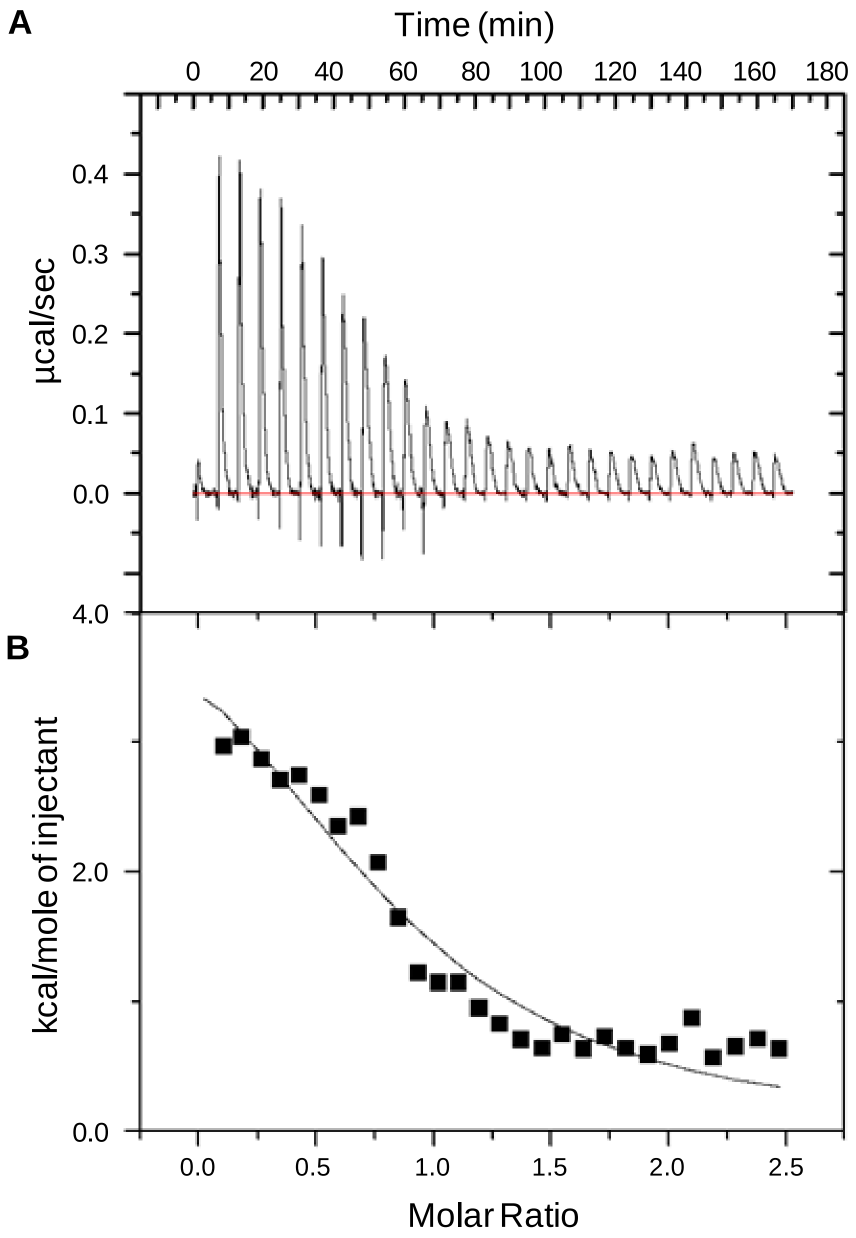


**Supplementary Figure 14: Isothermal titration calorimetry of *Pf*ISN1$_{\mathbf{D172N}}$ with IMP.** (**A**)Raw data of injection profile, corrected for heat of dilution of IMP. (**B**)Normalized heat of binding for each ligand injection.

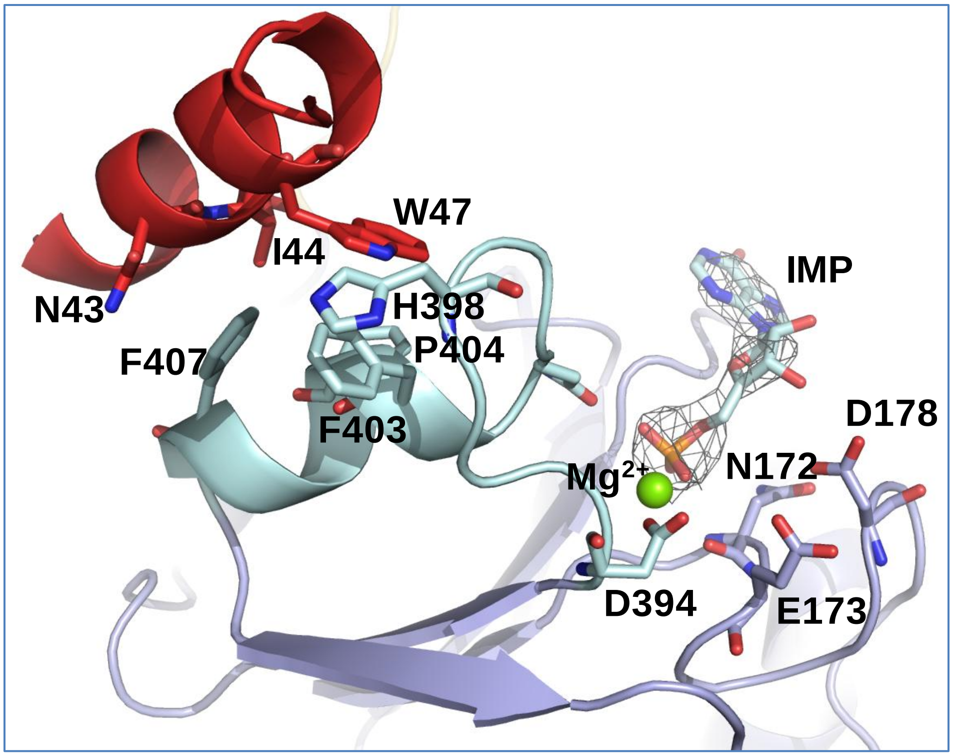


**Supplementary Figure 15: Intra-subunit stabilization of α-helix D402-F407 (cyan) by the NTRD α1 helix (red).** The *2Fo-Fc* electron-density map (grey mesh) is contoured at 1σ. Hydrophobic contacts contribute to the structuring and the stabilization of α-helix D402-F407.

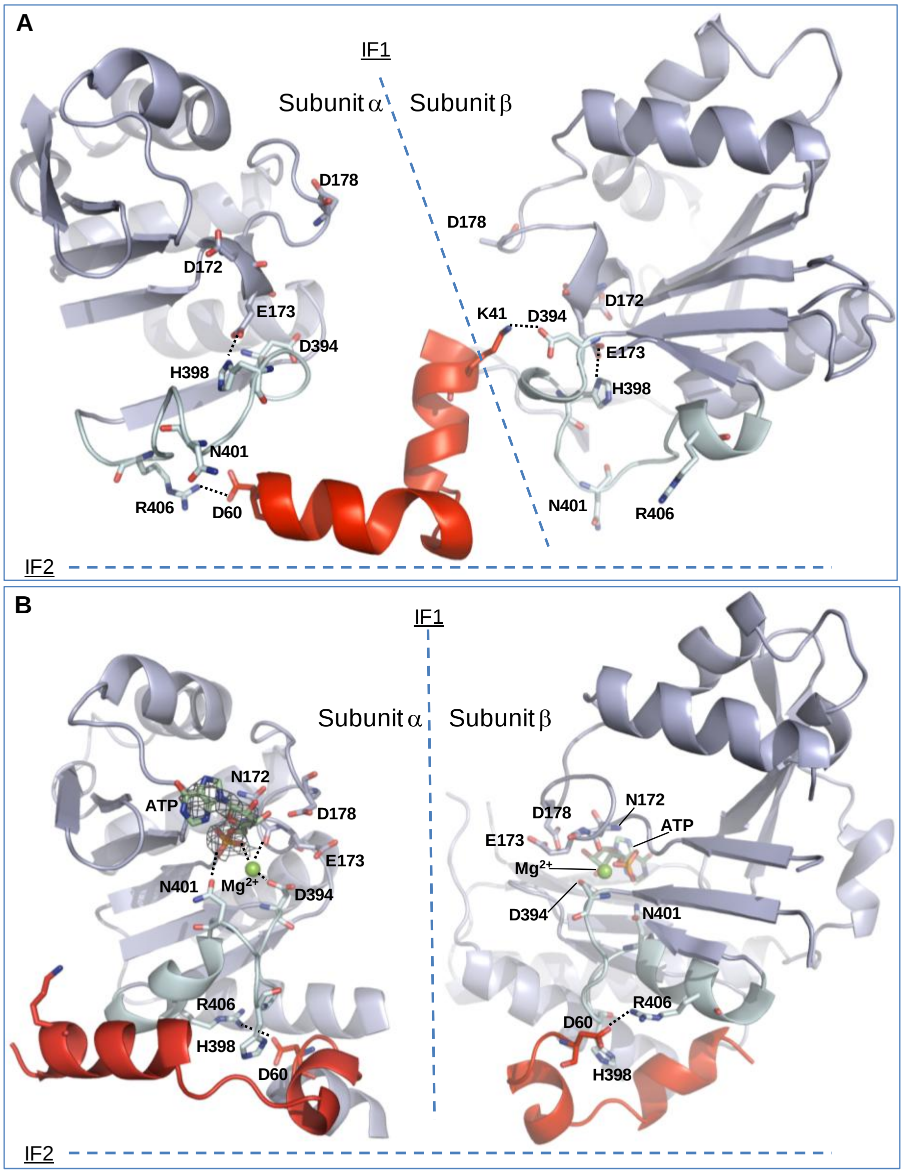


**Supplementary Figure 16: NTRD conformational change upon IMP binding** (**A**)In *Pf*ISN1-Apo, the subunit α NTRD is stabilized by intermolecular and intramolecular salt-bridges D394-K41 and D60-R406 (**B**)In *Pf*ISN1$_{D172N}$-IMP, the intermolecular salt-bridge D394-K41 is broken and NTRDs in subunits α and β are stabilized only by the intramolecular salt-bridge D60-R406. IF1, interface 1, IF2, interface 2. The *2Fo-Fc* electron-density map (dark grey mesh) is contoured at 1σ.

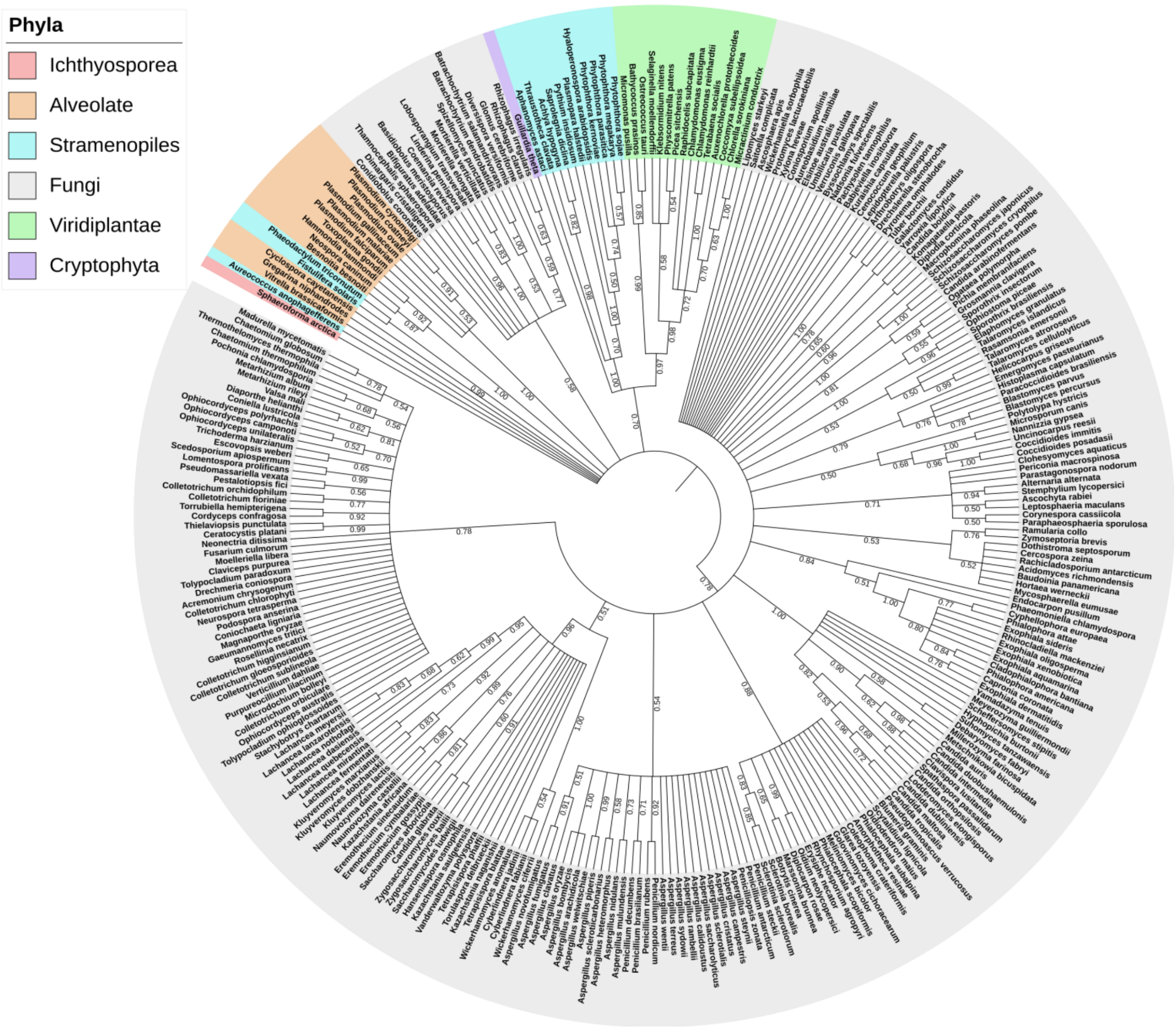


**Supplementary Figure 17: Distribution of ISN1 in the living kingdom.** Maximum likelihood tree for ISN1 sequences. Bootstrap support values for internal nodes are shown with values below 60% eliminated. Clades corresponding to major kingdoms/phyla are distinctively colored.

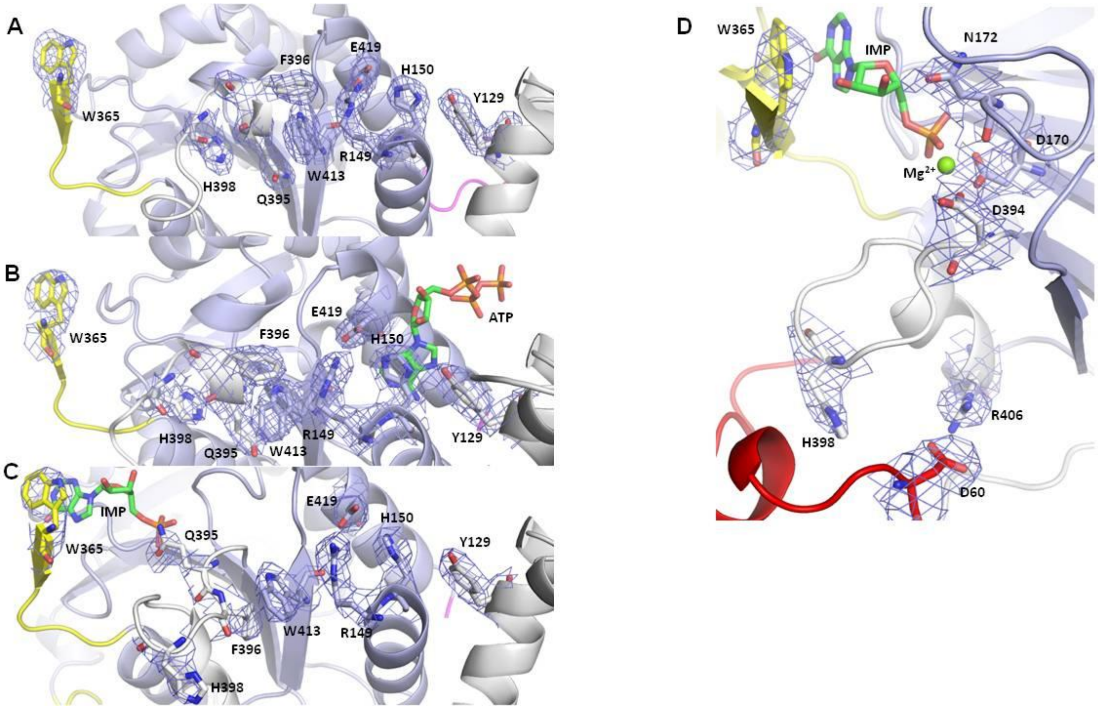


**Supplementary Figure 18: Electron density map of the main side chain residues involved in the catalysis and the structural reorganization of *Pf*ISN1.** Long range reorganization of ISN1 during the course of the catalysis in (**A**)Apo enzyme, (**B**)ATP bound enzyme and (**C**)IMP bound enzyme. (**D**)Organization of the IMP binding site. The *2Fo-Fc* electron-density maps (blue mesh) are contoured at 1σ.

## Supplementary Table 1. X-ray diffraction data collection and refinement statistics

| Structure-ID | *Pf*ISN1_SeMet-ATP | *Pf*ISN1-Apo | *Pf*ISN1-ATP | *Pf*ISN1$_{D172N}$-IMP | *Pf*ISN1-ΔC10 | *Pf*ISN1$_{D172N}$-ΔN30-IMP | *Pf*ISN1-ΔN59 |
|---|---|---|---|---|---|---|---|
| Data collection | | | | | | | |
| Beamline (ESRF) | ID29 | ID23-1 | ID30A | ID23-2 | ID30B | ID30B | ID30-A3 |
| Wavelength (Å) | 0.979 | 0.973 | 0.967 | 0.872 | 0.976 | 1.000 | 0.967 |
| Space group | $P6_522$ | $P2_1$ | $P6_522$ | $P2_1$ | $P6_522$ | $P2_1$ | $P4_22_12$ |
| Cell dimensions | | | | | | | |
| a, b, c (Å) | 210.2 210.2 105.8 | 149.0 204.1 149.2 | 210.7 210.7 106.2 | 108.3 204.6 115.9 | 211.9 211.9 105.1 | 107.8 203.9 115.3 | 140.7 140.7 87.0 |
| α, β, γ (°) | 90 90 120 | 90 90.02 90 | 90 90 120 | 90 113.2 90 | 90 90 120 | 90 113.4 90 | 90 90 90 |
| Resolution range (Å) | 47.1 - 3.7 | 48.3 - 2.6 | 47.4 - 2.8 | 47.7 - 3.4 | 19.9 - 3.7 | 49.7 - 3.5 | 29.6 - 3.0 |
| Total reflections | 590962 | 668774 | 607927 | 228074 | 288982 | 369099 | 492245 |
| Unique reflections | 27949 | 250293 | 34615 | 63502 | 15185 | 57015 | 18036 |
| $R_{meas}$ (%) | 14.5 (94.3) | 11.8 (69.4) | 10.2 (144.5) | 18.0 (101.6) | 40.4 (181.3) | 39.2 (128.6) | 14 (91.1) |
| $CC_{1/2}$ (%) | 99.9 (93.5) | 99.6 (81.9) | 100.0 (77.0) | 99.4 (54.8) | 99.8 (79.3) | 99.4 (76.7) | 99.9 (95.6) |
| I/σ(I) | 21.1 (5.5) | 7.5 (2.0) | 24.5 (2.3) | 7.8 (1.6) | 6.5 (2.5) | 3.9 (2.0) | 20.8 (4.6) |
| Multiplicity | 21 | 3 | 18 | 4 | 19 | 6 | 27 |
| Completeness (%) | 99.9 | 91.7 | 99.9 | 99.5 | 99.1 | 99.1 | 99.9 |
| No. mol. /asymm. unit | 2 | 16 | 2 | 8 | 2 | 8 | 2 |
| Refinement | | | | | | | |
| $R_{work}/R_{free}$ (%) | | 22.9/25.3 | 20.0/24.5 | 19.6/24.7 | 23.3/27.1 | 23.6/27.9 | 25.2/27.9 |
| No. atoms | | 52441 | 6327 | 23482 | 6169 | 23618 | 5179 |
| Protein | | 51136 | 6275 | 23275 | 6136 | 23414 | 5144 |
| Ligand/ion | | / | 43 | 204 | 18 | 204 | / |
| Water | | 1305 | 9 | 3 | 15 | / | 35 |
| Average B-factor ($Å^2$) | | 57.9 | 84.9 | 84.2 | 165.3 | 140.4 | 85.3 |
| Protein | | 58.1 | 84.6 | 84.1 | 165.3 | 140.3 | 85.3 |
| Ligand/ion | | / | 122.6 | 92.6 | 188.3 | 155.0 | / |
| Water | | 47.2 | 60.4 | 39.8 | 137.3 | / | 81.3 |
| r.m.s.d. | | | | | | | |
| Bond lengths (Å) | | 0.002 | 0.005 | 0.002 | 0.003 | 0.002 | 0.002 |
| Angles (°) | | 0.484 | 0.72 | 0.50 | 0.57 | 0.45 | 0.49 |
| Ramachandran | | | | | | | |
| Favored (%) | | 95.6 | 95.5 | 94.3 | 94.4 | 94.5 | 94.6 |
| Allowed (%) | | 4.3 | 4.6 | 5.1 | 5.3 | 5.3 | 5.2 |
| Outliers (%) | | 0.1 | 0.0 | 0.1 | 0.4 | 0.3 | 0.2 |

All diffraction data were collected on single crystals.

‡$R_{free}$, $R_{factor}$ calculated from 5% of the data excluded from refinement.

## Supplementary Table 2. SAXS data collection and scattering derived parameters

| | |
|---|---|
| Data-collection parameters | |
| Instrument | ESRF - BM29 |
| Beam geometry | 250 μm × 150 μm |
| Wavelength (Å) | 1,5418 |
| $q$ range ($nm^{-1}$) | 0.025 - 5 |
| Exposure time (min) | 45 |
| Concentration range ($mg\ ml^{-1}$) | ~13 |
| Temperature (K) | 283 |
| Structural parameters | |
| $I(0)$ ($cm^{-1}$) (from Guinier) | 4.2 ± 0.1 |
| $R_g$ (Å) (from Guinier) | 43 ± 0.9 |
| $D_{max}$ (Å) | 140 ± 9 |
| Molecular-mass determination | |
| Molecular mass [from $I(0)$] (Da) | 223,000 ± 3,500 |
| Molecular mass [from $V_c$] (Da) | 210,000 ± 2,290 |
| Calculated monomeric $M_r$ from sequence | 56 000 |
| Software employed | |
| Primary data reduction | ISPYB |
| Data processing | DATASW and Primus |
| *Ab initio* analysis | DAMMIN |
| Validation and averaging | DAMAVER |
| Rigid-body modeling | AllosMod-FoXS |
| Computation of model intensities | AllosMod-FoXS |
| Three-dimensional graphics representations | Chimera |

**Supplementary Table 3. Possible substrates tested**

| **Category** | **Compound**[a] | **No.** |
|---|---|---|
| Nucleoside mono-, di- and tri-phosphates | IMP, AMP, GMP, XMP, dAMP, dIMP, UMP, TMP, CMP, 3'AMP, succinyl AMP, cAMP, ADP, GDP, adenosine 3,5,-diphosphate, ATP, GTP, CTP, ITP | 19 |
| Glycerol phosphates | 2-PG, 3-PG, α-GP, β-GP, glycerophosphocholine | 5 |
| Sugar phosphates | PRPP, fructose-1,6-bisphosphate, glucose-1-phosphate, glucose-6-phosphate, mannose-6-phosphate, ribose-1-phosphate, ribulose-5-phosphate, erythrose-4-phosphate | 8 |
| Glycolytic intermediates | PEP, DHAP, 2,3-BPG | 3 |
| Amino acid phosphates | O-phospho-l-tyrosine, O-phospho-l-serine | 2 |
| Inositol phosphates | D-myo-inositol-1-phosphate, D-myo-inositol-4-phosphate, phosphoinositide mix, D-myo-inositol-3-phosphate, D-myo-inositol-1,4-diphosphate, D-myo-inositol-1,4,5-triphosphate | 6 |
| Vitamins and cofactors | NAD, NADP, NAM, NMN, NaMN, FMN, PLP | 7 |
| Other phosphates | 2-phosphoglycolic acid, phosphonoaceticacid, 3-deoxy-2-keto 6-phosphogluconic acid, Phosphoethanolamine, l-α-phosphatidylcholine, lysophosphatidylcholine, phosphocholine | 7 |
| Synthetic compounds | PNPP, PAPP, PNPS, phenyl phosphate | 4 |
| | **TOTAL** | **61** |

[a]All compounds were screened in 50 mM Tris-HCl, pH 8.0, 30 mM $MgCl_2$. The concentration of D-myo-inositol-1-phosphate, D-myo-inositol-4-phosphate was 5 mM whereas the concentration of all other compounds was 10 mM. Assay conditions are as described in Methods. All assays were performed in duplicate and repeated twice.

**Supplementary Table 4. List of modulators**

| | | | |
|---|---|---|---|
| ATP | dCTP | l-α-phosphatidylcholine[a] | choline chloride |
| TMP | GTP | D-myo-inositol-4-phosphate[a] | D-myo-inositol-3-phosphate |
| 2,3-BPG | NMN | mannose-6-phosphate | glucose-6-phosphate |
| dATP | dTTP | Phosphoinositide mix[a, b] | D-myo-inositol-1,4,5-triphosphate |
| UMP | GMP | myo-inositol | O-phospho-L-choline |
| ITP | | D-myo-inositol-1-phosphate[a] | l-α-glycerophosphocholine |
| CMP | | O-phosphoethanolamine | lysophosphatidylcholine[a] |
| dGTP | | D-myo-inositol-1,4-diphosphate | TOTAL = 28 |

All compounds were screened in 50 mM Tris-HCl, pH 8.0, 30 mM $MgCl_2$. The concentration of modulators used was 2 mM with IMP fixed at 10 mM. Assay conditions are as described in Methods.

[a]Concentration of IMP used was 5 mM.

[b]Catalog number P6023, Sigma Aldrich

*****All assays were performed in duplicate and repeated twice.

## Supplementary Table 5. List of oligonucleotide primers*

| Primer name | Primer sequence (5' to 3') |
|---|---|
| D170N stage I | GATTTATTAACAGATAT**C**GCTGACGAAACG |
| D170N stage II | GATTTATTAACATTTAATGCTGACGAAACG |
| D172N stage I | ACATTTGATGATAT**C**GAAACGCTATATCCG |
| D172N stage II | ACATTTGATGCTAACGAAACGCTATATCCG |
| D172A forward | GATTTATTAACATTTGATGCTGCCGAAACGCTATATCCGGATG |
| D172A reverse | CATCCGGATATAGCGTTTCGGCAGCATCAAATGTTAATAAATC |
| D170N-D172N forward | GTTTAGATTTATTAACATTTAATGCTAACGAAACGCTATATCCGGATG |
| D170N-D172N reverse | CATCCGGATATAGCGTTTCGTTAGCATTAAATGTTAATAAATCTAAAC |
| T7 forward | TAATACGACTCACTATAGGG |
| T7 reverse | TAATACGACTCACTATAGGG |
| gene-specific FP | CGGGATCCAAGAATTTGGACATAAATACATTCGATAATATTGAAGATATTCC |
| gene-specific RP | GGAAGCTTTTATTGATTTTCATATAAAACTTCCGGAATAAATGATTTTATG |
| K41L forward | GATAGAAATGTTATGAATTCAGATATGTTAAAAAATATTGTTCAGTGGAATAG |
| K41L reverse | CTATTCCACTGAACAATATTTTTTAACATATCTGAATTCATAACATTTCTATC |
| H150V forward | CAACATTCAATGAAGTTAGGGTTATACTTAATCTTGCTCAAATTTTG |
| H150V reverse | CAAAATTTGAGCAAGATTAAGTATAACCCTAACTTCATTGAATGTTG |
| Y176L forward | GATGCTGACGAAACGCTATTACCGGATGGTCATGATTTTAATG |
| Y176L reverse | CATTAAAATCATGACCATCCGGTAATAGCGTTTCGTCAGCATC |
| D178V forward | GACGAAACGCTATATCCGGTTGGTCATGATTTTAATG |
| D178V reverse | CATTAAAATCATGACCAACCGGATATAGCGTTTCGTC |
| R218L forward | GCAGAAAAATACCAAAAACTATTAGAGAATTTGTTAAAATATTTTTC |
| R218L reverse | GAAAAATATTTTAACAAATTCTCTAATAGTTTTTGGTATTTTTCTGC |
| D363V forward | CATTTAATGGAGGACAGGTTTTATGGGTAGACGTTG |
| D363V reverse | CAACGTCTACCCATAAAACCTGTCCTCCATTAAATG |
| W365L forward | GGATTTATTGGTAGACGTTGGTAATAAAGCGG |
| W365L reverse | CGTCTACCAATAAATCCTGTCCTCCATTAAATG |
| W365Y forward | GGAGGACAGGATTTATATGTAGACGTTGGTAATAAAG |
| W365Y reverse | CTTTATTACCAACGTCTACATATAAATCCTGTCCTCC |
| W365F forward | GGAGGACAGGATTTATTCGTAGACGTTGGTAATAAAG |
| W365F reverse | CTTTATTACCAACGTCTACGAATAAATCCTGTCCTCC |
| D367V forward | GACAGGATTTATGGGTAGTCGTTGGTAATAAAGCG |
| D367V reverse | CGCTTTATTACCAACGACTACCCATAAATCCTGTC |
| D394V forward | GAAATGTTGTCATATCGGTGTTCAGTTCTTACACTCAG |
| D394V reverse | CTGAGTGTAAGAACTGAACACCGATATGACAACATTTC |
| Q395L forward | GTCATATCGGTGATCTGTTCTTACACTCAGGAAATG |
| Q395L reverse | CATTTCCTGAGTGTAAGAACAGATCACCGATATGAC |
| F396L forward | CATATCGGTGATCAGTTATTACACTCAGGAAATGATTTTC |
| F396L reverse | GAAAATCATTTCCTGAGTGTAATAACTGATCACCGATATG |
| H398V forward | CATATCGGTGATCAGTTCTTAGTCTCAGGAAATGATTTTCC |
| H398V reverse | GGAAAATCATTTCCTGAGACTAAGAACTGATCACCGATATG |
| D402V forward | GTTCTTACACTCAGGAAATGTTTTTCCAACAAGATTTTGTAG |
| D402V reverse | CTACAAAATCTTGTTGGAAAAACATTTCCTGAGTGTAAGAAC |

| | |
|---|---|
| F403A forward | CTTACACTCAGGAAATGATGCTCCAACAAGATTTTGTAGTTTAA |
| F403A reverse | TTAAACTACAAAATCTTGTTGGAGCATCATTTCCTGAGTGTAAG |
| F403L forward | CAGTTCTTACACTCAGGAAATGATTTACCAACAAGATTTTGTAGTTT |
| F403L reverse | AAACTACAAAATCTTGTTGGTAAATCATTTCCTGAGTGTAAGAACTG |
| F403Y forward | GTTCTTACACTCAGGAAATGATTATCCAACAAGATTTTGTAGTTTAAC |
| F403Y reverse | GTAAACTACAAAATCTTGTTGGATAATCATTTCCTGAGTGTAAGAAC |
| R406L forward | CAGGAAATGATTTTCCAACATTATTTTGTAGTTTAACATTATG |
| R406L reverse | CATAATGTTAAACTACAAAATAATGTTGGAAAATCATTTCCTG |
| W413L forward | GTAGTTTAACATTATTGGTTAGCAACCCTCAAG |
| W413L reverse | CTTGAGGGTTGCTAACCAATAATGTTAAACTAC |
| ΔN30 | CGGGATCCGATAGAAATGTTATGAATTCAGATATGAAAAAAAATATTGTTC |
| ΔN59 | CGGGATCCAGTTTGATCATGTTTCTTGTAGAAATATTTAGATCTCTTTTTGTATCC |
| ΔC10 | GGAAGCTTTTATGATTTTATGTTTAAATGCATTATACTCTTTAAGCATGCTTTAG |
| PbCEN FP | TAAAAAATTTTATAAAACATAGGGGGATCCATGAAGAATTTGGACATAAATACA TTCGATAATATTG |
| GFP RP | CATATGATCTGGGTATCTCGCAAAGCATTG |
| PfCEN FP | TAAATACCTAATAGAAATATATCACCTAGGATGAAGAATTTGGACATAAATACA TTCGATAATATTGAAG |
| Pfisn1e3 FP | GGCCCCTTTCCGCGGGGAGGACTAGTTTGTTCAGTGGAATAGTCGATATAGCTA C |
| Pfisn1e5 RP | TTTTAATTTTTTTTACAAAATGCTTAAGTTAATAAATCTAAACCTTCTTCAAGGG AC |

*All oligonucleotide primers were custom synthesized at Sigma-Aldrich, India.

## Supplementary references


1. Altschul, S.F. et al. Gapped BLAST and PSI-BLAST: a new generation of protein database search programs. *Nucleic Acids Res* **25**, 3389-402 (1997).
2. Edgar, R.C. MUSCLE: multiple sequence alignment with high accuracy and high throughput. *Nucleic Acids Res* **32**, 1792-7 (2004).
3. Gouet, P., Courcelle, E., Stuart, D.I. & Metoz, F. ESPript: analysis of multiple sequence alignments in PostScript. *Bioinformatics* **15**, 305-8 (1999).
4. Kumar, S., Stecher, G. & Tamura, K. MEGA7: Molecular Evolutionary Genetics Analysis Version 7.0 for Bigger Datasets. *Mol Biol Evol* **33**, 1870-4 (2016).
5. Trager, W. & Jensen, J.B. Human malaria parasites in continuous culture. *Science* **193**, 673-5 (1976).
6. Fivelman, Q.L. et al. Improved synchronous production of *Plasmodium falciparum* gametocytes *in vitro*. *Mol Biochem Parasitol* **154**, 119-23 (2007).
7. Gupta, S., Schulman, S. & JP., Vanderberg. Stage-dependent toxicity of N-acetyl-glucosamine to *Plasmodium falciparum*. *J Protozool* **32**, 91-95 (1985).
8. Iwanaga, S., Kato, T., Kaneko, I. & Yuda, M. Centromere plasmid: a new genetic tool for the study of *Plasmodium falciparum*. *PLoS One* https://doi.org/10.1371/journal.pone.0033326 (2012).
9. Vaughan, A.M. et al. A transgenic *Plasmodium falciparum* NF54 strain that expresses GFP-luciferase throughout the parasite life cycle. *Mol Biochem Parasitol* **186**, 143-7 (2012).
10. Iwanaga, S. et al. Functional identification of the Plasmodium centromere and generation of a Plasmodium artificial chromosome. *Cell Host Microbe* **7**, 245-55 (2010).
11. Das, S., Lemgruber, L., Tay, C.L., Baum, J. & Meissner, M. Multiple essential functions of *Plasmodium falciparum* actin-1 during malaria blood-stage development. *BMC Biol* **15**, 70 (2017).
12. Ribaut, C. et al. Concentration and purification by magnetic separation of the erythrocytic stages of all human Plasmodium species. *Malar J* **7**, 45 (2008).
13. Schindelin, J. et al. Fiji: An Open-Source Platform for Biological-Image Analysis. *Nat Methods* **9**, 676-82 (2012).
14. Schneider, C.A., Rasband, W.S. & Eliceiri, K.W. NIH Image to ImageJ: 25 years of image analysis. *Nat Methods* **9**, 671-75 (2012).
15. Janse, C.J., Ramesar, J. & Waters, A.P. High-efficiency transfection and drug selection of genetically transformed blood stages of the rodent malaria parasite *Plasmodium berghei*. *Nat Protoc* **1**, 346-56 (2006).
16. Tonkin, C.J. et al. Localization of organellar proteins in *Plasmodium falciparum* using a novel set of transfection vectors and a new immunofluorescence fixation method. *Mol Biochem Parasitol* **137**, 13-21 (2004).
17. Shenoy, A.R. & Visweswariah, S.S. Site-directed mutagenesis using a single mutagenic oligonucleotide and DpnI digestion of template DNA. *Anal Biochem* **319**, 335-6 (2003).
18. Heckman, K.L. & Pease, L.R. Gene splicing and mutagenesis by PCR-driven overlap extension. *Nat Protoc* **2**, 924-32 (2007).

19. Beyer, H.M. et al. AQUA Cloning: A Versatile and Simple Enzyme-Free Cloning Approach. *PLoS One* https://doi.org/10.1371/journal.pone.0137652 (2015).
20. Bradford, M.M. A rapid and sensitive method for the quantitation of microgram quantities of protein utilizing the principle of protein-dye binding. *Anal Biochem* **72**, 248-54 (1976).
21. Chen, P.S.J., Toribara, T.Y. & Warner, H. Microdetermination of Phosphorus. *Anal. Chem.* **28**, 1756-1758 (1956).
22. Leskovac, V. Comprehensive enzyme kinetics. 243-282 (Kluwer Academic/Plenum Publishers, New York., 2003).
23. Terwilliger, T.C. et al. Decision-making in structure solution using Bayesian estimates of map quality: the PHENIX AutoSol wizard. *Acta Crystallogr D Biol Crystallogr* **65**, 582-601 (2009).
24. Afonine, P.V. et al. Towards automated crystallographic structure refinement with phenix.refine. *Acta Crystallogr D Biol Crystallogr* **68**, 352-67 (2012).
25. Emsley, P. & Cowtan, K. Coot: model-building tools for molecular graphics. *Acta Crystallogr D Biol Crystallogr* **60**, 2126-32 (2004).
26. Shkumatov, A.V. & Strelkov, S.V. DATASW, a tool for HPLC-SAXS data analysis. *Acta Crystallogr D Biol Crystallogr* **71**, 1347-50 (2015).
27. Schneidman-Duhovny, D., Hammel, M., Tainer, J.A. & Sali, A. FoXS, FoXSDock and MultiFoXS: Single-state and multi-state structural modeling of proteins and their complexes based on SAXS profiles. *Nucleic Acids Res* **44**, W424-9 (2016).
28. Franke, D. et al. ATSAS 2.8: a comprehensive data analysis suite for small-angle scattering from macromolecular solutions. *J Appl Crystallogr* **50**, 1212-1225 (2017).
29. Scheres, S.H.W. RELION: Implementation of a Bayesian approach to cryo-EM structure determination. *J Struct Biol* **180**, 519-30 (2012).
30. Bakan, A., Meireles, L.M. & Bahar, I. ProDy: protein dynamics inferred from theory and experiments. *Bioinformatics* **27**, 1575-7 (2011).
31. Holm, L. & Laakso, L.M. Dali server update. *Nucleic Acids Res* **44**, W351-5 (2016).
32. Pettersen, E.F. et al. UCSF Chimera-a visualization system for exploratory research and analysis. *J Comput Chem* **25**, 1605-12 (2004).
