# Supplementary material for: Structure and catalytic regulation of Plasmodium falciparum IMP specific nucleotidase
Source: Nat Commun. 2020 Jun 26;11:3228. doi: 10.1038/s41467-020-17013-x (PMC7320144; doi:10.1038/s41467-020-17013-x)
Supplement: Supplementary file 3 — Description of Additional Supplementary Information [file 41467_2020_17013_MOESM3_ESM.pdf]

Description of Additional Supplementary Files

File Name: Supplementary Movie 1
Description: 3D morphing of *Pf*ISN1 from Apo- to IMPbound conformations illustrating the tetramer reorganization.
